# Osmotic stress induces formation of both liquid condensates and amyloids by a yeast prion domain

**DOI:** 10.1016/j.jbc.2024.107766

**Published:** 2024-09-12

**Authors:** Anastasia V. Grizel, Natalia A. Gorsheneva, Jonathan B. Stevenson, Jeremy Pflaum, Florian Wilfling, Aleksandr A. Rubel, Yury O. Chernoff

**Affiliations:** 1 School of Biological Sciences, Georgia Institute of Technology, Atlanta, Georgia, USA; 2 Laboratory of Amyloid Biology and Department of Genetics and Biotechnology, St Petersburg State University, St Petersburg, Russia; 3 Mechanisms of Cellular Quality Control, Max Planck Institute of Biophysics, Frankfurt, Germany

**Keywords:** amyloid, biomolecular condensate, liquid–liquid phase separation, prion, stress response, translation termination factor, *Ogataea methanolica*, *Saccharomyces*, Sup35, yeast

## Abstract

Liquid protein condensates produced by phase separation are involved in the spatiotemporal control of cellular functions, while solid fibrous aggregates (amyloids) are associated with diseases and/or manifest as infectious or heritable elements (prions). Relationships between these assemblies are poorly understood. The *Saccharomyces cerevisiae* release factor Sup35 can produce both fluid liquid-like condensates (*e.g*., at acidic pH) and amyloids (typically cross-seeded by other prions). We observed acidification-independent formation of Sup35-based liquid condensates in response to hyperosmotic shock in the absence of other prions, both at increased and physiological expression levels. The Sup35 prion domain, Sup35N, is both necessary and sufficient for condensate formation, while the middle domain, Sup35M antagonizes this process. Formation of liquid condensates in response to osmotic stress is conserved within yeast evolution. Notably, condensates of Sup35N/NM protein originated from the distantly related yeast *Ogataea methanolica* can directly convert to amyloids in osmotically stressed *S. cerevisiae* cells, providing a unique opportunity for real-time monitoring of condensate-to-fibril transition *in vivo* by fluorescence microscopy. Thus, cellular fate of stress-induced condensates depends on protein properties and/or intracellular environment.

A variety of proteins contain intrinsically disordered regions, IDRs, capable of undergoing liquid–liquid phase separation, LLPS (1, 2). LLPS produces reversible dynamic condensates (designated here and further as “liquid”) sometimes maturing into gel-like assemblies. LLPS-based assemblies, termed coacervates, are proposed to have played an important role in the origin of life (3). Liquid and/or gel-like protein condensates are also frequently detected in living cells, although it has been questioned whether they originate solely from concentration-dependent LLPS (4). Indeed, interactions between β-strands, generated within low complexity IDRs were proposed to play a role in the condensate formation (5). Nevertheless, protein condensates may act as membraneless organelles and are implicated in many biologically important processes, such as protection against stresses, intracellular trafficking and compartmentalization, and regulation of gene expression (6, 7). Many disease-related proteins also produce liquid condensates (8). Condensate formation by some proteins can be promoted by stresses, such as hyperosmotic stress (9).

In addition to liquid or gel-like condensates, some IDR-containing proteins form solid cross-β fibrillar assemblies, termed amyloids (10). Amyloids are associated with numerous devastating human and animal diseases, including the fatal and widespread Alzheimer’s disease, Parkinson’s disease, Huntington’s disease, amyotrophic lateral sclerosis, and infectious “prion” diseases, also termed transmissible spongiform encephalopathies (11, 12, 13, 14). These diseases can spread within the organism (and in the case of infectious diseases, between organisms) *via* the process of “nucleated polymerization,” where preexisting fibrillary polymer immobilizes a monomeric protein with the amyloid-forming region of the same sequence, converting it into a prion form *via* the formation of an intermolecular β-sheet (15, 16). In contrast to liquid biomolecular condensates, pathogenic amyloids are generally irreversible. Some amyloids or amyloid-like assemblies are also implicated in adaptive roles, such as scaffolding melanin polymerization, cell-to-cell/cell-to-substrate attachment, or long-term neuron potentiation and memory (17, 18). One and the same protein may potentially form both liquid (and/or gel-like) condensates and solid amyloid fibrils, and existing evidence (primarily *in vitro*) indicates that condensates may convert into amyloids under certain circumstances (19, 20, 21, 22, 23, 24, 25). However, the overall relationship between these two types of assemblies is poorly understood.

Fungi, including *Saccharomyces* yeast*,* possess a variety of proteins capable of forming self-perpetuating assemblies (fungal prions), that are heritable in a non-Mendelian fashion and may control detectable phenotypic traits (26, 27). Many (26), although not all (28, 29, 30) known fungal prions are based on amyloids. Some fungal prions are clearly pathogenic to their host (31), while other prions are proposed to play adaptive roles (29, 30, 32, 33). The impact of pathogenic prions on fungal biology could be significant, as yeast cells maintain complex antiprion machinery involving chaperones, components of proteolytic pathways, and sorting factors (34). Yeast prions or prion-like aggregates could be induced by stresses and maintain stress memory (35, 36, 37). Powerful genetic tools make yeast an excellent model for studying general mechanisms and biological implications of prions and amyloids (38).

*Saccharomyces cerevisiae* prion [*PSI*^*+*^], formed by translation termination factor Sup35 is arguably the best-studied fungal prion to date (26, 38). The Sup35 protein is composed of N-terminal NQ-rich IDR-containing prion domain (PrD) involved in amyloid formation (Sup35N), middle domain containing clusters of charged residues (Sup35M), and C-proximal functional release factor region (Sup35C). [*PSI*^*+*^] is readily detectable in specially engineered strains due to increased readthrough of stop codons (nonsense-suppression). Sup35 or Sup35N/NM overproduction (39, 40), as well as various stresses, including hyperosmotic stress (35) were shown to promote [*PSI*^*+*^] formation, but these effects were usually significant only in the presence of another prion, such as a prion form of Rnq1 protein, [*PIN*^*+*^], capable of cross-seeding [*PSI*^*+*^] formation (41, 42, 43). Recombinant Sup35NM is typically used to study amyloid formation *in vitro*, while fluorophore-tagged Sup35 or Sup35N/NM derivatives are employed to monitor aggregation by fluorescence microscopy (FM) in yeast cells (44). Fluorophore-tagged Sup35 or Sup35N/NM forms amyloid assemblies in *S. cerevisiae* cells containing [*PSI*^*+*^] prion, or upon overproduction in initially [*psi*^*-*^] cells with other preexisting aggregates, such as [*PIN*^*+*^] (44, 45, 46). Mature prions appear as dot-like puncta, while filamentous intermediates (“ribbons” or “rings”) can be seen in the process of *de novo* [*PSI*^*+*^] generation in [*PIN*^*+*^] cells (45, 46, 47). In response to acidic stress, nonprion Sup35 also forms reversible liquid condensates (48). Conflicting reports identify either Sup35NM (48) or Sup35C (49) as the major determinant of acidification-dependent condensate formation. *In vitro*, formation of liquid Sup35NM condensates can also be promoted by crowding agents such as PEG 6000, and these condensates may either facilitate or antagonize formation of the Sup35NM amyloids depending on protein concentration (50).

Here, we specifically address the impact of osmotic stress on Sup35 assemblies. Our data show osmotic stress promotes formation of reversible condensates driven by the Sup35N domain in an acidification-independent manner *in vivo* and that these condensates may convert into fibrillar amyloids upon prolonged stress, depending on the evolutionary origin of the Sup35N/NM region.

## Results

### Formation of Sup35N/NM assemblies in [psi^-^ pin^-^] yeast cells

**Figure 1:**
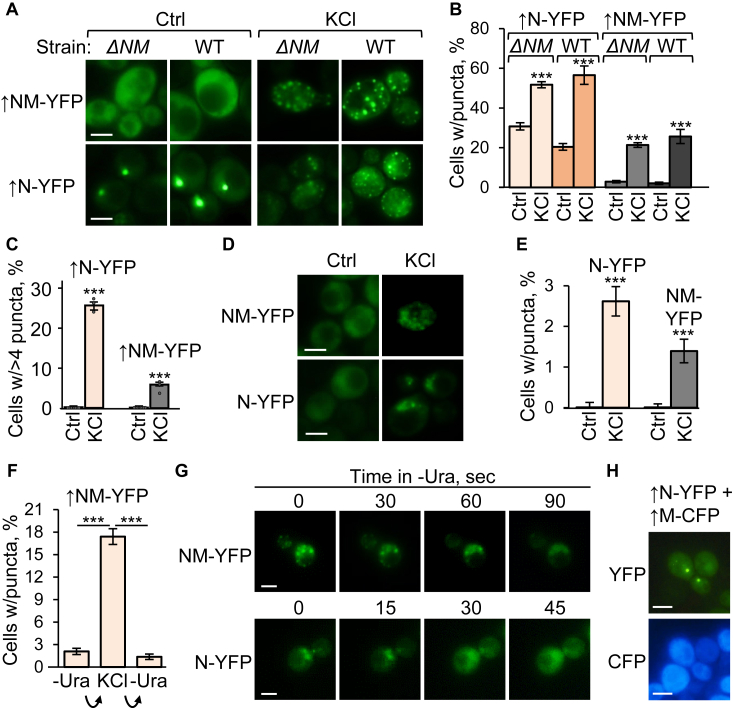
**Formation of condensates by fluorophore-tagged Sup35-based constructs.***A*, images and *B*, percentages—Detection of puncta in [*psi*^-^*pin*^-^] WT and *sup35-ΔNM* cells expressing Sup35N-YFP or Sup35NM-YFP at high levels in medium with 100 μM CuSO_4_, either in the absence of (Ctrl) or after 5 min in the presence of 1 M KCl. For each strain/plasmid combinations, one representative culture is shown; for numbers of repeats and variation between repeats, see Table S1. *C*, proportions of cells with more than four puncta per cell in cultures with excess Sup35N/NM-YFP before (Ctrl) and after short (5–15 min) osmotic stress with 1 M KCl in [*psi*^-^*pin*^-^] *sup35-ΔNM* strain. *D*, images and *E*, percentages—Detection of condensates in cells of the [*psi*^-^*pin*^-^] *sup35-ΔNM* strain expressing Sup35N-YFP or Sup35NM-YFP at moderate levels without extra CuSO_4_. *F*, dissolution of condensates of excess Sup35N/NM-YFP (induced by 10-min incubation in 1 M KCl) following the shift to synthetic plasmid-selective (-Ura) medium without a stressor for 10 min. *G*, the dissolution of biomolecular condensates formed by overproduced *Saccharomyces cerevisiae* Sup35N/NM-YFP constructs in the [*pin*^*-*^*psi*^*-*^] *sup35-ΔNM S. cerevisiae* cells as detected by time-lapse microscopy after moving cells from 1 M KCl solution to the –Ura synthetic medium. *H*, Sup35M-CFP does not colocalize with Sup35N-YFP and does not inhibit formation of Sup35N-YFP condensates *in trans* in [*psi*^*-*^*pin*^*-*^] *sup35-ΔNM* cells grown for 24 h in the presence of 100 μM CuSO_4_. Error bars indicate the standard errors of proportion, SEs. Scale bars represent 5 μm. ∗∗∗ indicates statistically significant (*p* < 0.001) differences from control according to Fisher’s exact test.

Our data confirms that overproduction of fluorophore-tagged Sup35NM (*e.g.,* Sup35NM-YFP) from the copper-inducible (*P*_*CUP1*_) promoter in *S. cerevisiae* cells, grown in the presence of increased levels of CuSO_4_ and lacking any preexisting prions ([*psi*^*-*^ *pin*^*-*^]) typically produces diffuse fluorescence (Fig. 1, *A*–*B* and Table S1), with very rare cells containing large fluorescent agglomerates of unknown nature. Notably, we found that, while most [*psi*^*-*^ *pin*^*-*^] cells overproducing Sup35N-YFP also show diffuse fluorescence, some produce globular puncta, termed henceforth “biomolecular condensates” (Fig. 1, *A*–*B* and Table S1). Short-term (2–15 min) treatment with osmotic stressors, such as 1 M or 2 M KCl, 1 M or 2 M NaCl, 2 M glycerol, 2 M glucose, 1.5 M or 2.7 M sorbitol, induced condensate formation by both Sup35N-YFP and Sup35NM-YFP (Figs. 1, *A*–*B*, S1, *A*–*C*, and Tables S1, S2), and increased both the proportion of condensate-containing cells (Figs. 1, *A*–*B*, S1, *A*–*B* and Tables S1, S2) and number of condensates per cell in the case of Sup35N-YFP (Fig. 1*C* and Table S3). Condensates were formed by constructs with different fluorophores, such as YFP or GFP (Fig. S1, *E*–*F* and Table S4), and detected in both cells containing or lacking (*sup35-ΔNM*) the chromosomal Sup35NM-coding region (Fig. 1, *A*–*B* and Table S1). Condensates were also detected in osmotically stressed [*psi*^*-*^ *pin*^*-*^] cells expressing Sup35N-YFP or Sup35NM-YFP at moderate levels in standard synthetic medium containing 3 μM CuSO_4_ (Figs. 1, *D*–*E*, S1, *B* and *D* and Tables S5, S6), indicating that a high-level overproduction is not required for condensate formation. Importantly, condensates induced by osmotic stress were reversible within 1 to 2 min after transferring cells to medium without the osmotic stressor (Fig. 1, *F*–*G* and Table S7). YFP protein that was not fused to Sup35N or NM did not produce condensates in response to osmotic stress (Table S2).

Taken together, this data shows that the presence of the Sup35N region is sufficient for the ability to form a condensate. Comparison between Sup35N and NM indicates that Sup35M antagonizes condensate formation *in cis*; however such antagonism is not observed when Sup35M-CFP is coexpressed with Sup35N-YFP *in trans* (Fig. 1*H*).

### Inclusion of full-length Sup35 protein into biomolecular condensates at physiological protein levels

**Figure 2:**
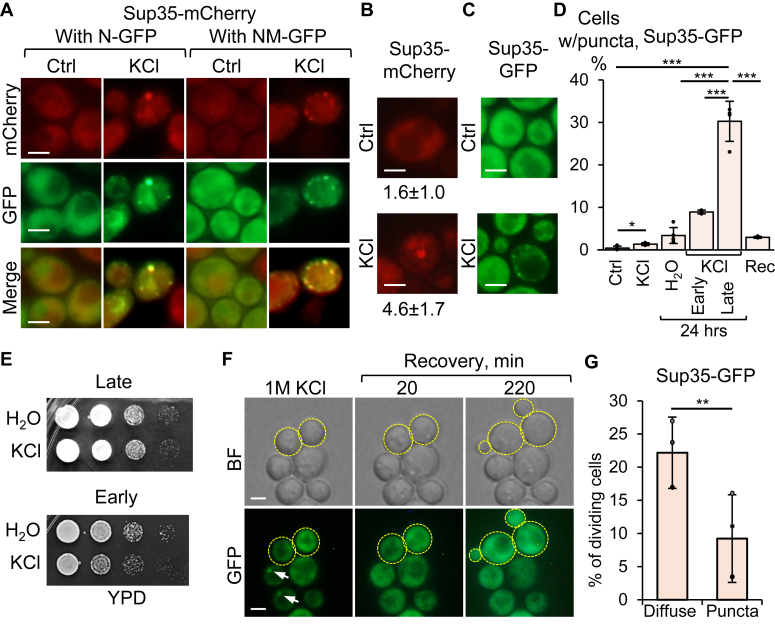
**Formation of condensates by full-length Sup35 protein and their correlations with cell viability and proliferation.***A*, inclusion of full-length Sup35 protein into Sup35N/NM-GFP biomolecular condensates generated at background levels of Cu^++^ after 5 to 15 min in 1 M KCl, in comparison to media without stressor (Ctrl). *B*, induction of condensates after short exposure to 1 M KCl in cells containing only the mCherry-tagged chromosomal *SUP35* gene under the endogenous promoter. Average % with SEs are indicated (see Table S8). *C*, condensate formation by chromosomally encoded full-length Sup35-GFP after short exposure to 1 M KCl. *D*, quantitation of condensate formation by chromosome-encoded full-length Sup35-GFP in various conditions (Ctrl, no stress; KCl, 1 M KCl for 5–30 min or 24 h, where indicated; Rec, 10 min recovery in fresh SC media after 24-h osmotic stress; early and late refer to early exponential; 6 h or late exponential/stationary; and 24 h cultures, respectively). See Table S9 for numbers. *E*, hyperosmotic stress is more toxic for early exponential cultures, relative to late exponential/stationary cultures of the strain expressing chromosomally encodedSup35-GFP; decimal serial dilutions of cells incubated in H_2_O or 1 M KCl for 24 h are shown. *F*, images and *G*, diagram—Delay of poststress budding in cells that initially had condensates relative to cells with diffuse fluorescence, as observed over 5 h of incubation, using time-lapse microscopy (see Table S10 for frequencies). Dividing cells are highlighted with *yellow dashed lines*, condensates are indicated with *arrows*. Scale bars on all images correspond to 5 μm.

Osmotic stress-induced condensates of Sup35N/NM-GFP, detected at moderate expression levels (corresponding to background levels of Cu^++^ present in synthetic media) in [*psi*^*-*^ *pin*^*-*^] cells bearing the chromosomal copy of full-length *SUP35* tagged with mCherry (under the endogenous *P*_*SUP35*_ promoter), incorporated full-length Sup35-mCherry protein (Fig. 2*A*). Abundance of Sup35 protein or Sup35N/NM-based constructs *per se* was not increased by osmotic stress (Fig. S2, *A* and *B*).

Furthermore, formation of condensates by full-length Sup35-mCherry (Fig. 2*B* and Table S8) or Sup35-GFP (Fig. 2, *C*–*D* and Table S9) proteins, produced from the chromosomal gene under the endogenous *P*_*SUP35*_ promoter was detected in osmotically stressed [*psi*^*-*^ *pin*^*-*^] cells lacking any plasmid-based Sup35-derived constructs. While the frequency of cells with condensates of chromosome-encoded Sup35-GFP was relatively low after short-term (15–30 min) osmotic stress, it increased significantly after long-term (24 h) incubation in the presence of an osmotic stressor, compared to cells incubated in water (Fig. 2*D* and Table S9). This data shows that osmotic stress promotes formation of condensates by full-length Sup35 protein at physiological protein levels.

### Viability and recovery of yeast cells after osmotic stress

Early exponential cultures produced condensates at a lower frequency than late exponential or early stationary cultures (Fig. 2*D* and Table S9). Notably, prolonged osmotic stress was more toxic to early exponential cultures than late exponential cultures (Fig. 2*E*). Although Sup35-GFP condensates were solubilized quickly after return to nonstress conditions (Fig. 2*D*), a significant fraction of cells that previously contained condensates exhibited resumption of budding compared to cells not containing condensates (Fig. 2, *E*–*F* and Table S10). This indicates that condensates may play a functional role in and/or serve as biological indicator of some processes related to recovery from stress.

### Formation of biomolecular condensates during osmotic stress is not due to acidification

**Figure 3:**
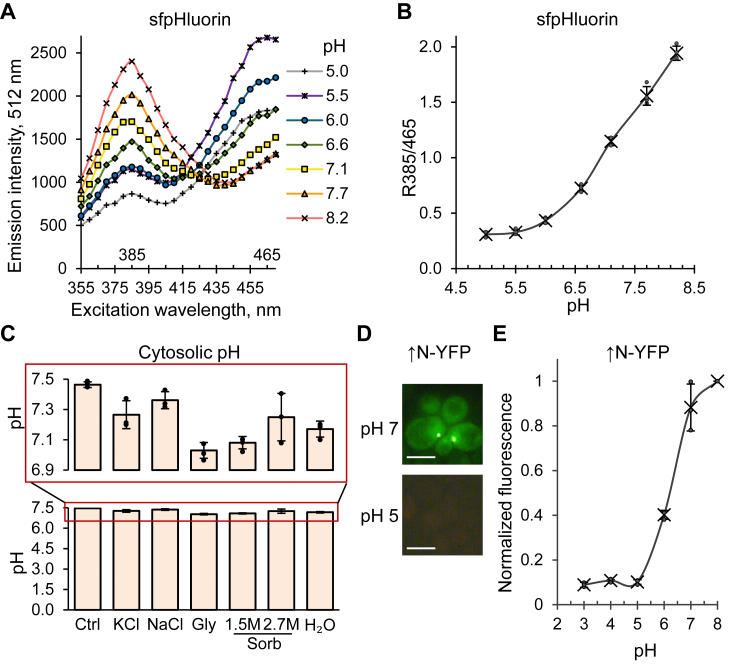
**Measuring intracellular pH and impact of osmotic stress on pH.***A*, pH dependence of excitation spectra of sfpHluorin expressed in *Saccharomyces cerevisiae* [*pin*^*-*^*psi*^*-*^] *sup35-ΔNM* strain. Cells were permeabilized with digitonin and subsequently resuspended in a citric acid/Na_2_HPO_4_ buffer with pH values ranging from 5.0 to 8.2. The emission intensity was recorded at (512 ± 20) nm. The assay was performed in four biological replicates. One representative replicate is shown. For numbers, see Dataset S1. *B*, pH calibration curve of sfpHluorin. Ratio R385/465 for the wavelength (512 ± 20) nm was calculated *via* dividing emission intensity at excitation wavelength of 385 nm by emission intensity at excitation wavelength of 465 nm. Calibration data has been corrected for cell autofluorescence. Individual data points from four replicates, means (indicated by *crosses*), and SDs are shown. *C*, osmotic stress does not make intracellular pH acidic, as measured using pH-sensitive colorimetric fluorescent protein sfpHluorin. Three independent cultures were used for each treatment. 1 M concentrations were used for KCl and NaCl; Gly and Sorb correspond to glycerol (2 M) and sorbitol, respectively. The *upper part* of the diagram at neutral pH is zoomed in and shown within a *red frame*. *D*, YFP used in this work is sensitive to acidic intracellular pH. Cells overproducing Sup35N-YFP were permeabilized with digitonin and subsequently resuspended in a citric acid/Na_2_HPO_4_ buffer at pH 5 or pH 7. Fluorescence was detected using two fluorescent filters simultaneously (YFP and RFP) to differentiate specific YFP fluorescent signals from background autofluorescence detectable in both channels. Scale bars represent 5 μm. *E*, dependence of YFP fluorescence on intracellular pH. Cells overproducing Sup35N-YFP were permeabilized with digitonin and subsequently resuspended in a citric acid/Na_2_HPO_4_ buffer at pH values ranging from 3 to 8. The emission intensity was recorded at 535 ± 20 nm. Individual data points from three replicates, means (indicated by *crosses*), and SDs are shown.

As acidic pH stress is known to cause formation of Sup35 biomolecular condensates (48), we employed sfpHluorin, a pH-sensitive fluorescent protein (51) to investigate whether or not osmotic stress acidifies the intracellular environment of the *S. cerevisiae* strains used in our work. Calibration data linking sfpHluorin fluorescence to pH has been composed as shown on Fig. 3, *A*–*B*, Table S11, and Dataset S1. Our measurements (Fig. 3*C* and Table S12) indicate that intracellular pH is only slightly decreased by osmotic stress and remains above neutral. Notably, fluorescence of YFP protein that was used as a fluorophore in many of our experiments has been reported to be sensitive to acidification (52). Our data (Fig. 3, *D*–*E* and Table S13) confirms that YFP fluorescence is decreased in acidic pH and cannot be detected at pH levels at or below 5, which were previously reported to induce condensate formation during acidic pH stress (48). These results, in combination with our observation that Sup35M region, containing a putative pH-sensing cluster (48) is not required for condensate formation during osmotic stress (see above, Fig. 1) clearly demonstrate that the formation of Sup35-based assemblies during hyperosmotic stress is not due to acidification of the intracellular environment.

### Analysis of the fluidity patterns of Sup35N-YFP and Sup35NM-YFP condensates

**Figure 4:**
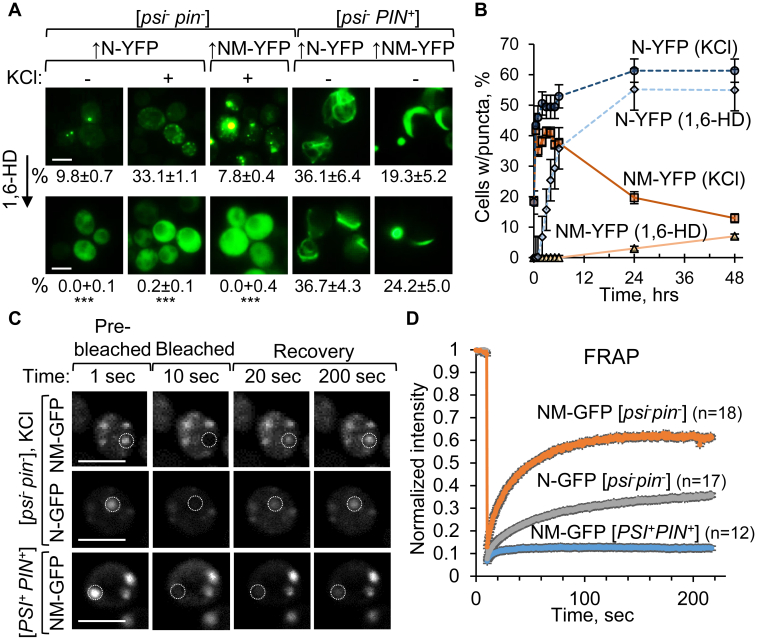
**Fluidity of biomolecular condensates.***A*, sensitivity ([*psi*^-^*pin*^-^]) and resistance (in [*psi*^-^*PIN*^+^]) to 1,6-HD of the assemblies formed by excess Sup35N/NM-YFP in *sup35-ΔNM* cells. Incubation was for 5 min (KCl) and 10 min (1,6-HD). Numbers indicate average percentages (with SEs) of cells with condensates; ∗∗∗ indicates statistically significant (*p* < 0.001) differences from 1,6-HD–untreated control according to Fisher’s exact test. *B*, accumulation of 1,6-HD–resistant puncta of overproduced Sup35N/NM-YFP after prolonged osmotic stress. *C*, images and *D*, graph - FRAP of condensates formed by overproduced Sup35N/NM-GFP in the [*psi*^-^*pin*^-^] *sup35-ΔNM* strain after 30-min 1 M KCl treatment, in comparison to the amyloids formed by overproduced Sup35NM-GFP in the [*PSI*^+^ *PIN*^+^] strain. *White circles* indicate bleached areas. Scale bars on all images correspond to 5 μm. See also Videos S3–S5, and Dataset S2. 1,6-HD, 1,6-to hexanediol; FRAP, fluorescence recovery after photobleaching; SE, standard error.

Biomolecular condensates formed by Sup35N/NM-YFP in [*psi*^*-*^ *pin*^*-*^] cells exhibited round shape, consistent with a liquid state (Videos S1 and S2), and were solubilized by 1,6-hexanediol (1,6-HD) (Fig. 4*A* and Table S14). 1,6-HD dissolves liquid droplets and some (but not all) gel-like assemblies; however, it does not affect solid assemblies (53, 54). Notably, a fraction of osmotic stress induced Sup35N-YFP condensates became 1,6-HD–resistant after prolonged incubation in the presence of 1 M KCl, and cells with such 1,6-HD–resistant puncta constituted a majority after 24 to 48 h of incubation (Fig. 4*B* and Table S15). In the case of Sup35NM-YFP, the proportion of cells with puncta significantly decreased after 24- to 48-h stress, however some remaining puncta also became resistant to 1,6-HD (Fig. 4*B* and Table S15). These data point to the possibility of “gelation” and/or “solidification” of some initially liquid condensates after prolonged stress.

Sup35NM-GFP puncta induced by short-term osmotic stress (1 M KCl) in [*psi*^*-*^ *pin*^*-*^] cells also exhibited rapid fluorescence recovery after photobleaching (FRAP), in contrast to solid amyloid puncta detected in the [*PSI*^*+*^] strains, which were essentially irrecoverable (Fig. 4, *C*–*D*, Videos S3–S4, and Dataset S2). This data confirms the fluid nature of Sup35NM-based biomolecular condensates. Notably, Sup35N-GFP condensates recovered faster than Sup35NM-GFP amyloids but slower than Sup35NM-GFP condensates in [*psi*^*-*^ *pin*^*-*^] (Fig. 4, *C*–*D*, Video S5, and Dataset S2). This indicates that fluid Sup35N-GFP condensates may undergo initial stages of conversion into a less mobile (possibly gel-like) state during the 30-90-min time period needed for the sample preparation and FRAP measurement.

**Figure 5:**
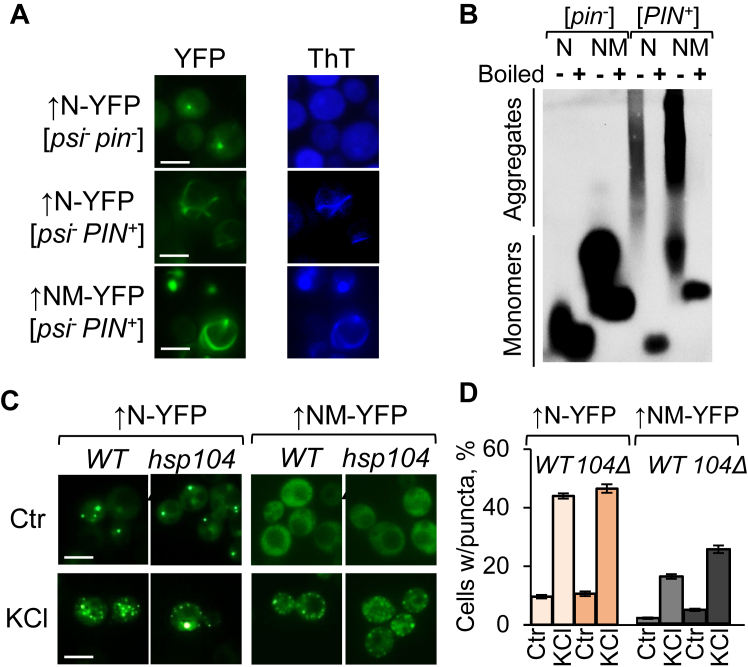
**Biomolecular condensates are not amyloids.***A*, ThT binds amyloid aggregates formed by overproduced Sup35N/NM-YFP in [*psi*^-^*PIN*^+^] cells, but not condensates formed by overproduced Sup35N-YFP in [*psi*^-^*pin*^-^] *sup35-ΔNM* cells. *B*, Sup35N/NM-YFP condensates produced in [*psi*^*-*^*pin*^*-*^] cells do not contain SDS-resistant polymers, in contrast to amyloids formed in isogenic [*PIN*^*+*^] *sup35-ΔNM* cells. Samples indicated by (+) were preboiled for 7 min to solubilize detergent-resistant aggregates. From 0.45 to 0.75 mg of total protein were loaded onto each lane. *C*, images and *D*, percentages—Dispensability of the Hsp104 chaperone for the formation and maintenance of Sup35N/NM-YFP condensates. Scale bars on all images correspond to 5 μm. ThT, thioflavin T.

The nonamyloid nature of Sup35N-based condensates formed in [*psi*^*-*^ *pin*^*-*^] cells without osmotic stress was confirmed by their inability to bind the amyloid-specific dye thioflavin T (ThT), in contrast to Sup35N/NM-YFP amyloids formed in [*PIN*^*+*^] cells (Fig. 5*A*). Unlike amyloids, Sup35N/NM-YFP condensates are solubilized by SDS without boiling, as detected by semidenaturing detergent agarose gel electrophoresis, SDD-AGE (Fig. 5*B*). Moreover, accumulation of Sup35N/NM-YFP condensates in [*psi*^*-*^ *pin*^*-*^] background occurred independently of the presence or absence of the chaperone protein Hsp104 (Fig. 5, *C*–*D* and Table S16), which is required for the propagation of amyloid-based Sup35 prions (26, 55). Overall, our results are consistent with the notion that stress-inducible condensates formed by Sup35-derived constructs represent nonamyloid liquid assemblies, some of which can convert into gel-like or solid state upon prolonged stress.

### Osmotic stress-induced condensate formation is conserved across diverse yeast species

**Figure 6:**
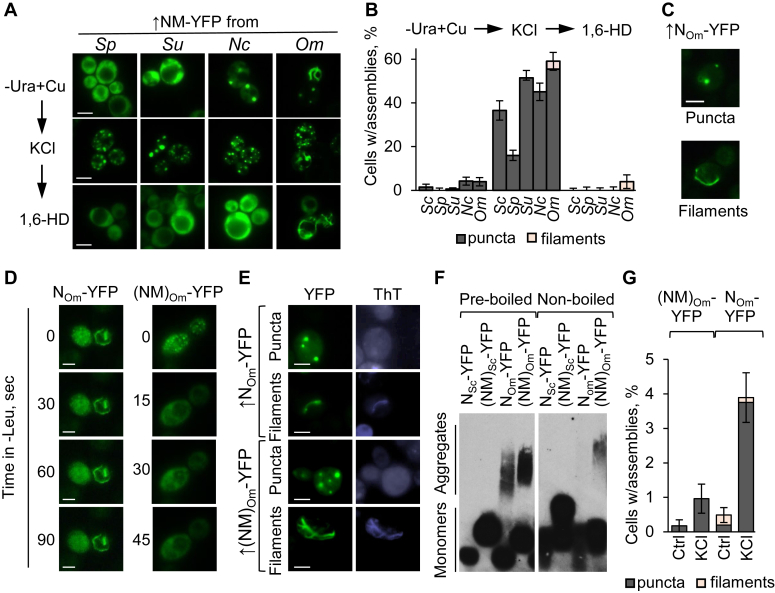
**Osmotic stress-induced phase separation of Sup35NM is evolutionarily conserved across various yeast species.***A*, images and *B*, percentages—Impact of overproduction (in the presence of 100 μM Cu^++^), osmotic stress (1 M KCl for 5 min), and 1,6-HD on assemblies of YFP-tagged Sup35NM domains from various yeast species in *Saccharomyces cerevisiae* [*psi*^-^*pin*^-^] *sup35-ΔNM* cells. Sc, Sp, Su, Nc, and Om correspond to *S. cerevisiae*, *Saccharomyces paradoxus*, *Saccharomyces uvarum*, *Naumovozyma castellii,* and *Ogataea methanolica*, respectively. Error bars indicate SEs. Scale bars represent 5 μm. *C*, examples of assemblies formed by *O. methanolica* Sup35N-YFP in [*psi*^*-*^*pin*^*-*^] *sup35-ΔNM* cells. *D*, the dissolution of biomolecular condensates formed by overproduced *O. methanolica* (Om) Sup35N/NM-YFP constructs (as shown) in the [*pin*^-^*psi*^-^] *sup35-ΔNM S. cerevisiae* cells as detected by time-lapse microscopy after moving cells from 1 M KCl solution to –Leu synthetic medium. Note that filamentous assemblies (as one seen in the case of N_Om_-YFP) are not dissolved. *E*, staining of filamentous Sup35(NM)_Om_-YFP and Sup35N_Om_-YFP aggregates in *sup35-ΔNM* [*pin*^-^] cells by ThT. *F*, detection of detergent-resistant Sup35(N/NM)_Om_-YFP polymers in *sup35-ΔNM* [*pin*^-^] cells by SDD-AGE. *G*, frequencies of cells filaments and puncta, observed with or without osmotic stress in [*psi*^*-*^*pin*^*-*^] *sup35-ΔNM* cultures, producing Sup35(N/NM)_Om_-YFP at moderate levels (after growth in the medium with background levels of Cu^++^). Scale bars on all images correspond to 5 μm. ThT, thioflavin T; SDD-AGE. semidenaturing detergent agarose gel electrophoresis; 1,6-HD, 1,6-to hexanediol.

The Sup35NM region exhibits a high rate of evolutionary divergence, compared to Sup35C (56, 57, 58). Since Sup35 LLPS in response to acidic stress is conserved in yeast evolution (48), we investigated whether formation of Sup35 condensates after overproduction and osmotic stress is also conserved. Indeed, osmotic stress induced formation of puncta in the *sup35-ΔNM* [*psi*^*-*^ *pin*^*-*^] *S. cerevisiae* strain overproducing YFP-tagged Sup35NMs from *Saccharomyces paradoxus* and *Saccharomyces uvarum,* which are species closely related to *S. cerevisiae* (Fig. 6, *A*–*B* and Table S17). Overproduced Sup35NM-YFP from *Naumovozyma castellii*, more distantly related to *S. cerevisiae*, produced puncta even in the absence of stress, with greater abundance after stress. In all cases, puncta were dissolved by 1,6-HD, indicating their liquid composition. Notably, YFP-tagged Sup35NM or Sup35N from *Ogataea* (formerly *Pichia*) *methanolica* (*Om*), which is the most diverged from *S. cerevisiae* among the tested species, produced both puncta and filamentous structures (ribbons or rings) upon overproduction in both *sup35-ΔNM* [*psi*^*-*^ *pin*^*-*^] *S. cerevisiae* cells (Fig. 6, *A*–*C* and Table S17) and in [*pin*^-^ *psi*^-^] *S. cerevisiae sup35Δ* cells bearing a chimeric gene *SUP35(NM)*_*Om*_*-C*_*Sc*_, with Sup35NM-coding region from *Ogataea methanolica* and Sup35C-coding region from *S. cerevisiae* (56) on a low-copy (*CEN*) plasmid (Fig. S3 and Table S13). Most puncta (but not filaments) were reversible and were sensitive to 1,6-HD (Fig. 6, *A*–*D* and Table S18). Abundances of Sup35(NM)_Om_-С_Sc_, Sup35N_Om_-YFP and Sup35(NM)_Om_-YFP proteins were not increased after either short (5 min) or prolonged (24 h) osmotic stress (Fig. S2, *C*–*D*), confirming that increased formation of condensates during stress is not due to increased protein abundance. Filamentous Sup35(N/NM)_Om_-YFP assemblies were stained by ThT (Fig. 6*E*) and contained detergent-resistant polymers detectable by SDD-AGE (Fig. 6*F*), confirming their amyloid composition. Notably, Sup35(N/NM)_Om_-YFP polymers were not completely solubilized by boiling, and Sup35N_Om_-YFP polymers were capable of entering the agarose gel only after boiling (Fig. 6*F*), indicating that these polymers are more temperature-resistant and, in the case of Sup35N_Om_-YFP, are probably larger than boiling-sensitive polymers formed by Sup35_Sc_ derivatives (Fig. 5*B*). Both puncta and filaments and induction of puncta by osmotic stress were also detected in cells producing Sup35N_Om_-YFP or Sup35(NM)_Om_-YFP at background levels of Cu^++^ (Fig. 6*G* and Table S19), albeit at lower frequencies than highly overproducing cells. Overall, our data indicate broad evolutionary conservation of Sup35’s ability to form osmotic stress-induced condensates and show that *O. methanolica* Sup35 is capable of forming both condensates and solid filamentous aggregates in the absence of other prions.

### Osmotic stress promotes the formation of [PSI^+^] prion in the absence of other prions

**Figure 7:**
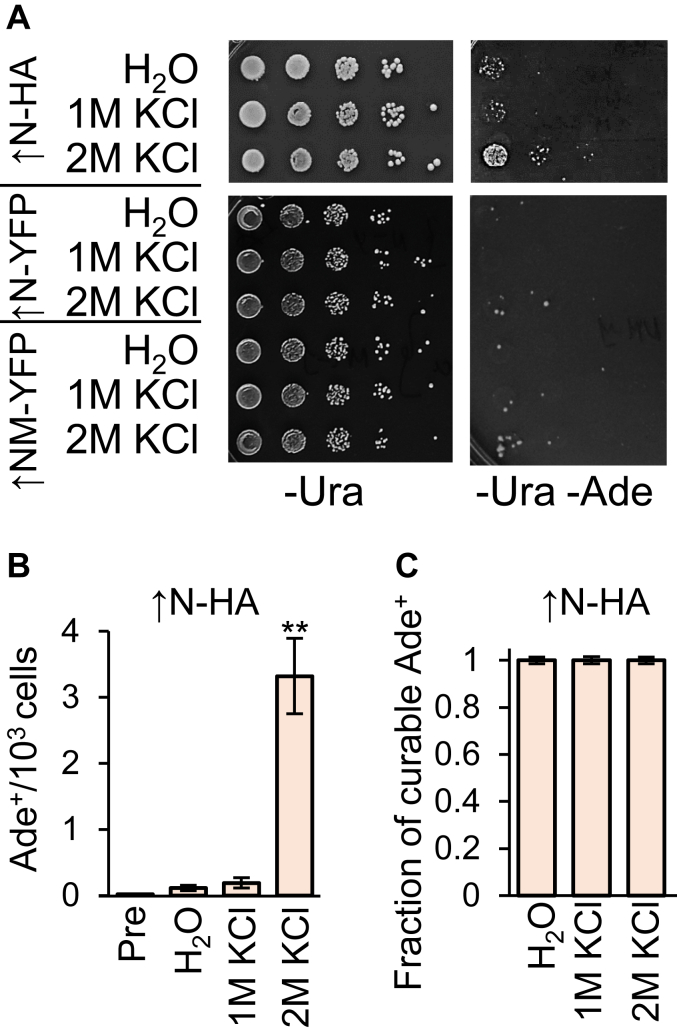
**Prion induction by osmotic stress in cells overproducing *Saccharomyces cerevisiae* Sup35N/NM-based constructs.***A*, image and *B*, quantitation—Effect of osmotic stress on [*PSI*^+^] prion formation in the *S. cerevisiae* [*psi*^-^*pin*^-^] strain overproducing Sup35N-HA, or Sup35N/NM-YFP proteins. Overproduction of Sup35N-HA and Sup35N/NM-YFP was induced by addition of 100 μM CuSO_4_ to the medium at 24 h prior to treatment. Decimal serial dilutions were spotted onto -Ura (for detection of plasmid-containing cells) and onto –Ura-Ade (for [*PSI*^+^] detection). ∗ and ∗∗ indicate statistically significant differences from H_2_O control (*p* < 0.05 and *p* < 0.01, respectively) according to paired *t* test. All experiments were performed in at least four replicates. Error bars show SDs. *C*, majority of Ade^+^ colonies induced by osmotic stress in the [*pin*^-^] *S. cerevisiae* strain overproducing Sup35N-HA are curable by GuHCl, indicating that they contain the [*PSI*^+^] prion. Error bars show SEs. GuHCL, guanidine hydrochloride; HA, hemagglutinin; SE, standard error.

It has previously been reported that in cells containing another prion, [*PIN*^*+*^], prolonged incubation with some osmotic stressors facilitates [*PSI*^*+*^] formation by a destabilized derivative of Sup35 protein with an increased number of oligopeptide repeats (35). Thus, we investigated whether osmotic stress promotes [*PSI*^*+*^] formation in the absence of [*PIN*^*+*^]. As *S. cerevisiae* [*PSI*^*+*^] seldom arises at normal protein levels in [*pin*^*-*^] backgrounds, these experiments employed a [*psi*^*-*^ *pin*^*-*^] *S. cerevisiae* strain that contained full-length *SUP35* on the chromosome and expressed hemagglutinin (HA)-tagged Sup35N from a copper-inducible *P*_*CUP1*_ promoter on a plasmid. We described previously (59) that overexpression of Sup35N-HA causes infrequent formation of adenine (Ade^+^) colonies ([*PSI*^*+*^]) in this [*pin*^*-*^] strain, which could be detected by growth on −Ade medium due to readthrough of the nonsense mutant *ade1-14* (UGA) reporter gene (26, 44). Notably, Ade^+^ formation in the presence of Sup35N-HA was significantly increased after 24-h incubation in the presence of 2 M KCl (Fig. 7, *A*–*B* and Table S20). Most Ade^+^ colonies generated in these conditions contained [*PSI*^*+*^] prions, as they were curable by guanidine hydrochloride, GuHCl (Fig. 7*C* and Table S21), an antiprion agent inhibiting the Hsp104 chaperone (60, 61). In contrast to Sup35N-HA, [*PSI*^*+*^] induction by osmotic stress was not detected in [*pin*^*-*^] cells overproducing Sup35N-YFP or Sup35NM-YFP (Fig. 7*A*).

**Figure 8:**
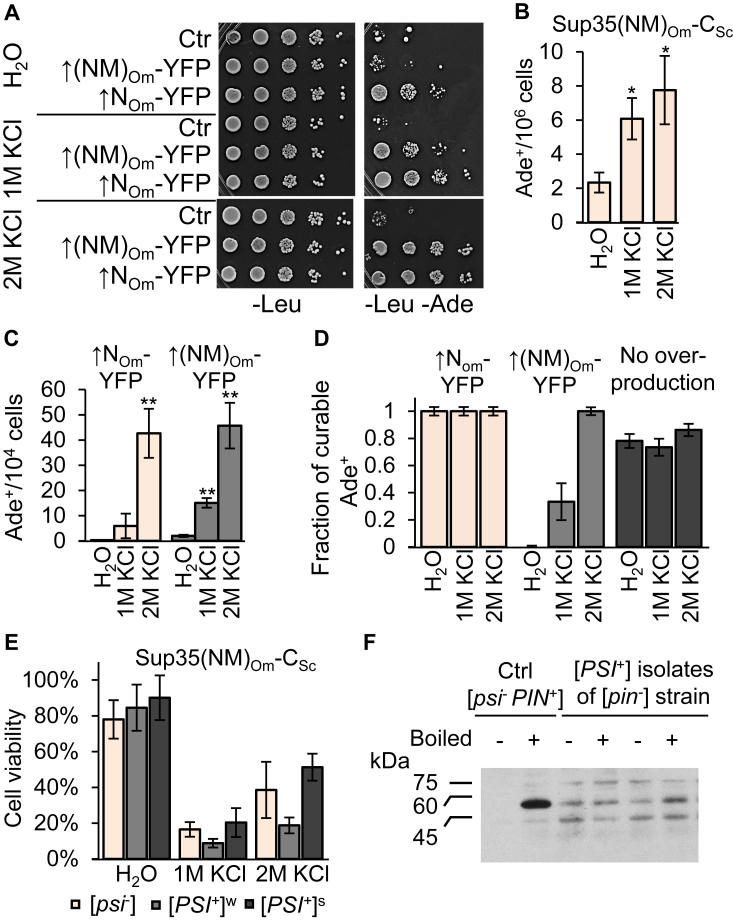
**Prion induction by osmotic stress in *Saccharomyces cerevisiae* [*psi***^**-**^***pin***^**-**^**] cells expressing chimeric Sup35(NM)**_**Om**_**-C**_**Sc**_**protein.***A*, images; *B* and *C*, quantitation—Induction of [*PSI*^+^] prion by osmotic stress in *S. cerevisiae* [*psi*^-^*pin*^-^] cells expressing chimeric Sup35(NM)_Om_-C_Sc_ protein and either lacking (*B*), or containing overexpressed (*C*)) *Ogataea methanolica SUP35N/NM-YFP* on a plasmid. On panel A, decimal serial dilutions were spotted onto -Leu (for detection of plasmid-containing cells) and, respectively, onto -Leu-Ade (for [*PSI*^+^] detection). Overproduction of Sup35N/NM-YFP was induced by addition of 100 μM CuSO_4_ to the medium at 24 h prior to treatment. Cells were incubated with an osmotic stressor for 24 h. Control (Ctrl) on panels A contained empty *LEU2* vector pRS315. Error bars show SDs; ∗ and ∗∗ indicate statistically significant differences from H_2_O control (*p* < 0.05 and *p* < 0.01, respectively) according to paired *t* test. All experiments were performed at least in four replicates. *D*, majority of Ade^+^ colonies induced by osmotic stress in the strain with Sup35(NM)_Om_-C_Sc_ protein either in the presence of excess Sup35(N/NM)_Om_-YFP or in the absence of any overproducing construct are curable by guanidine hydrochloride (GuHCl), indicating that they contain the [*PSI*^+^] prion. *E*, neither strong ([*PSI*^+^]^s^) nor weak ([*PSI*^+^]^w^) variant of the Sup35(NM)_Om_-C_Sc_ prion confers fitness advantage during osmotic stress. Five independent colonies of each prion variant were tested. *F*, the gel entry assay indicates the absence of the [*PIN*^+^] prion in [*PSI*^+^] colonies induced by osmotic stress in cells with Sup35(NM)_Om_-C_Sc_. Aggregated Rnq1 protein from the extracts of control [*PIN*^+^] cells does not enter SDS-PAGE without boiling (−), but is detected in large amount if a sample is preboiled (+). The unboiled and preboiled [*pin*^-^] samples show about the same amount of Rnq1 protein, but less than in case of [*PIN*^+^], due to increased proteolytic degradation during protein isolation in boiled and not boiled samples. Two representative examples out of 10 [*PSI*^+^] colonies tested are shown. Additional bands are typical for the Rnq1 samples in our hands and possibly represent either modified or partly degraded Rnq1 species or nonspecific activity of the Rnq1 antibody. Position and sizes of the molecular weight markers are indicated.

To further explore the impact of osmotic stress on prion formation, we employed *O. methanolica* Sup35-derived constructs that are capable of forming amyloid aggregates in *S. cerevisiae* cells lacking other prions (see above, Fig. 6). Formation of Ade^+^ colonies in the *S. cerevisiae* [*psi*^-^ *pin*^-^] strain producing the aforementioned chimeric Sup35(NM)_Om_-C_Sc_ protein (56) instead of endogenous Sup35 was increased at several fold after 24 h of incubation with 1 M or 2 M KCl (Fig. 8, *A*–*B* and Table S22). Excess Sup35N_Om_-YFP induced Ade^+^ formation in such a strain even without stress, while osmotic stress further increased [*PSI*^*+*^] formation in the presence of either excess Sup35N_Om_-YFP or excess Sup35(NM)_Om_-YFP (Fig. 8, *A* and *C* and Table S23). Most of the Ade^+^ colonies obtained in the presence of an osmotic stressor (with or without overproduction) were [*PSI*^*+*^], as the Ade^+^ phenotype was curable by GuHCl (Fig. 8*D*, and Tables S24, S25). Importantly, such [*PSI*^*+*^] derivatives did not exhibit increased survival during osmotic stress compared to [*psi*^*-*^] cells (Fig. 8*E* and Table S26), showing that an increase in [*PSI*^*+*^] frequency is not due to a selective advantage of [*PSI*^*+*^]. Notably, [*PSI*^*+*^] derivatives of the strain bearing the Sup35(NM)_Om_-C_Sc_ protein did not contain Rnq1 in a prion form ([*PIN*^*+*^]), showing that formation of [*PSI*^*+*^] was not due to prior induction of [*PIN*^*+*^] (Fig. 8*F*). Taken together, these results confirm that osmotic stress facilitates formation of the [*PSI*^*+*^] prion in the absence of other prions.

### Conversion of liquid condensates into amyloid filaments

**Figure 9:**
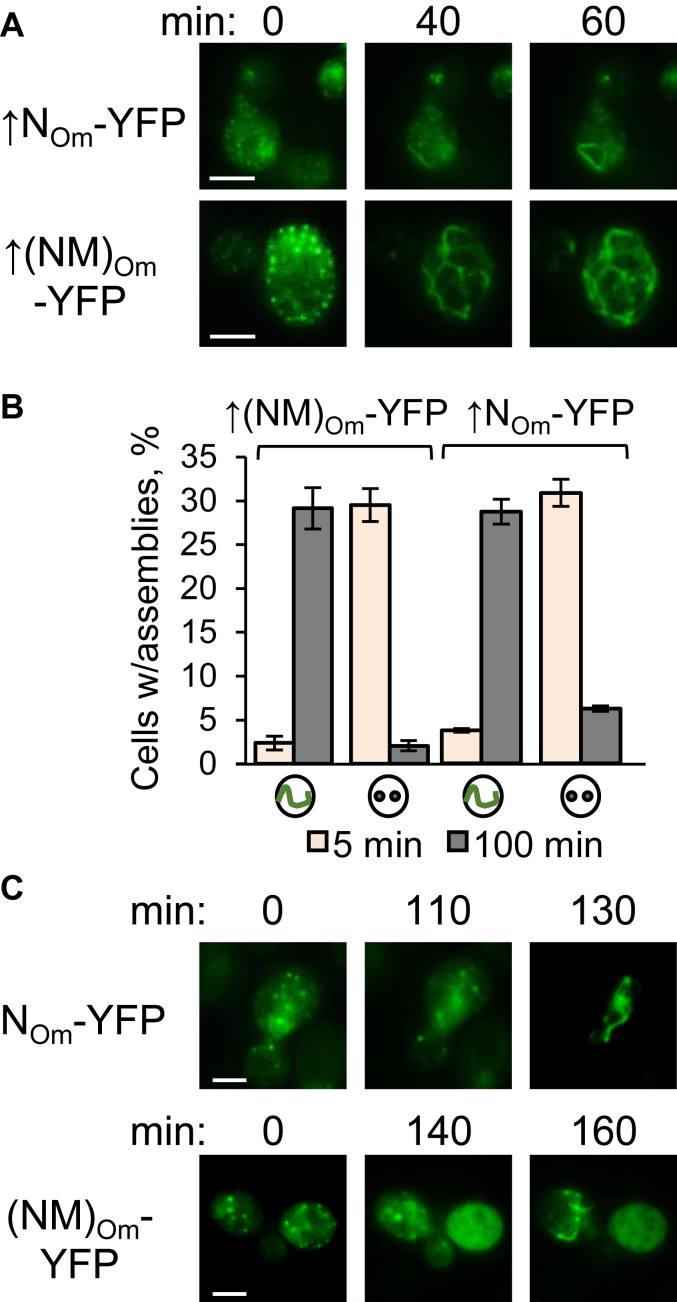
**Conversion of *Ogataea methanolica* Sup35N/NM-YFP from biomolecular condensates into filamentous amyloid formations.** Conversion of overproduced *A*, single-cell time lapse images and *B*, graph or nonoverproduced *C*, single-cell time lapse images. *O. methanolica* Sup35N/NM-YFP from condensates () into the filamentous amyloids () during osmotic stress (1 M KCl) in *Saccharomyces cerevisiae sup35-ΔNM* [*psi*^-^*pin*^-^] cells. See Videos S6–S9. Scale bars represent 5 μm.

Next, we studied the relationship between liquid condensates and filamentous amyloids, using a unique property of *O. methanolica* Sup35N/NM, which can produce both types of assemblies in [*pin*^*-*^] *S. cerevisiae* cells lacking preexisting prions. Forty four out of 87 tested individual *S. cerevisiae sup35-ΔNM* [*pin*^*-*^] cells with condensates of overproduced Sup35N_Om_-YFP, and all 62 tested cells with condensates of overproduced Sup35(NM)_Om_-YFP exhibited conversion of condensates into filamentous structures upon continuous incubation in the presence of osmotic stressor (1 M KCl), starting from 40 min and completed by 60 to 80 min of incubation, as detectable by time-lapse microscopy (Fig. 9*A*, and Videos S6–S7). Likewise, the overall proportion of cells with puncta was greatly decreased, while the proportion of cells with filaments was increased in cultures incubated with osmotic stressor for 100 min, compared to 5 min (Fig. 9*B* and Table S27). Conversion of Sup35(N/NM)_Om_-YFP condensates into filaments was also detected in *sup35Δ* [*pin*^*-*^] cells containing the chimeric *SUP35(NM)*_*Om*_*-C*_*Sc*_ gene (Fig. S4*A*), as well as in *sup35-ΔNM* [*pin*^*-*^] cells producing Sup35N_Om_-YFP or Sup35(NM)_Om_-YFP at moderate levels after growth in the medium with background levels of Cu^++^, albeit after a longer period of incubation in 1 M KCl (Fig. 9*C*, and Videos S8–S9), Condensate-to-filament conversion was also observed in the absence of stress (Fig. S4*B*), although only in a minor fraction of condensates (4 out of 12 individually monitored cells for Sup35N_Om_-YFP, and one out of four for Sup35(NM)_Om_-YFP), while most condensates were dissolved under these conditions. Apparently, stress counteracts condensate dissolution, but is not essential for amyloid formation. Overall, our data confirms that fluorophore-tagged *O. methanolica* Sup35N/NM condensates can directly convert into amyloids.

### Comparison of protein levels for various Sup35-derived constructs

As LLPS is frequently described as concentration-dependent processes, differences in protein levels could impact some results obtained in our work. To evaluate the potential impact of altered Sup35 concentrations, we carefully compared levels of various Sup35-derived constructs used in the experiments described above. Using inmmunoblotting followed by densitometry, we observed that *S. cerevisiae* Sup35N-YFP and Sup35NM-YFP constructs expressed under the *P*_*CUP1*_ promoter in the medium with background concentration of Cu^++^ are accumulated at similar levels to each other and at about 15- to 18-fold higher levels than endogenous full-length untagged Sup35 protein produced from chromosomal gene (Fig. S5, *A*–*D* and Table S28). Notably, *O. methanolica* Sup35N-YFP and Sup35NM-YFP constructs were produced from the *P*_*CUP1*_ promoter at background level of Cu^++^ in amounts similar to those of respective *S. cerevisiae* constructs (Fig. S5, *A*–*D* and Table S28), while full-length *S. cerevisiae* GFP-tagged Sup35 expressed from a chromosome copy under endogenous promoter was accumulated at only slightly (and not statistically significantly) lower level than endogenous untagged full-length Sup35 (Fig. S5, *B* and *D* and Table S28). As this was difficult to compare moderately and highly expressed YFP-tagged constructs by densitometry due to saturation of the signal, we employed fluorescence-based detection for these comparisons. Calibration of YFP fluorescence measurements confirmed their linear behavior within the desirable range (Fig. S5*E* and Table S29). Our measurements indicated that addition of 100 μM CuSO_4_ increased protein accumulation at 6- to 14-fold, depending on the construct (Fig. S5*F* and Table S30). Importantly, levels of highly overproduced *O. methanolica* Sup35N- or NM-YFP were consistently not higher (and in case of Sup35NM, somewhat lower) than the levels of the respective *S. cerevisiae* constructs produced in the same conditions, as confirmed by both immunoblotting (Fig, S5*A*) and fluorescence measurements (Fig. S5*F* and Table S30). Taken together, our data confirm that while increased protein levels indeed increase phase separation (especially during osmotic stress), they are not required for this process. We also demonstrate that the higher amyloid-forming propensity of the *O. methanolica* based constructs is not due to a relative increase in protein levels than respective *S. cerevisiae* constructs.

## Discussion

### Formation of stress-induced condensates by Sup35 derivatives

Previous data has indicated that Sup35NM-containing constructs undergo reversible LLPS *in vitro* and *in vivo* in response to acidification (48) or *in vitro* in the presence of a crowding agent such as PEG 6000 (50). Different studies point to either Sup35M region, containing charged clusters (48), or Sup35C region, interacting with other translation factors (49) as major modulators of Sup35 phase separation in response to acidic pH *in vivo*. Osmotic stress has been reported to produce solid globular Sup35-containing assemblies only in strains containing Sup35 in an amyloid (prion) form (62). While overproduced Sup35 PrD (Sup35N) exhibits a robust amyloid nucleation barrier in the absence of other prions, occasional LLPS-like condensation can be observed upon supersaturation (63). Liquid condensates have been proposed to represent an adaptive assembly pathway that is alternative to amyloid formation and is primarily regulated by pH changes *via* the charged C-proximal section of Sup35M (48).

Our data shows that overproduction of the Sup35N region in cells lacking preexisting prions results in accumulation of globular condensates, even in the absence of stress, while exposure to osmotic stressors promotes condensate formation by both Sup35N and Sup35NM constructs, both at high and moderate levels of expression, as well as by fluorophore-tagged full-length Sup35 expressed at physiological levels (Figs. 1 and 2). Globular shape, solubilization by 1,6-HD, and fast FRAP kinetics are consistent with LLPS as a potential mechanism of condensate generation (Fig. 4). Osmotic stress does not lead to acidification of the yeast cytosol, and at least one fluorophore employed by us, YFP, is not detectable by fluorescence at acidic pH, ruling out the possibility of that condensates are only detected in cells with lower pH (Fig. 3). Thus, condensate formation during hyperosmotic stress is not due to acidic pH. Facilitation of condensate formation by overproduction indicates that saturation of protein concentrations likely plays a role in the formation of condensates, while osmotic stress may exacerbate this process by producing a crowding effect in the yeast cytosol *via* water loss and cell shrinking. Cell-to-cell variations in protein levels may contribute to cell-to-cell variations in condensate accumulation. However, condensate formation cannot be explained solely by overproduction, because osmotic stress also induces condensate formation in a fraction of cells at moderate and even physiological levels of expression (Figs. 1, *D*–*E* and 2, *B*–*D*). This shows that condensate formation does not simply occur as a by-product of artificial overproduction and is indeed physiologically relevant. Local supersaturation at some intracellular locations potentially plays a role the condensation-promoting effect of some stress-induced proteins and/or other cellular components cannot be ruled out. Notably, condensate formation at physiological levels of Sup35 is significantly increased upon prolonged incubation in the presence of an osmotic stressor (Fig. 2, *C*–*D*), showing that not only protein concentration *per se* but also the duration of stress play an important role in the promotion of phase separation. It is possible that cell shrinkage is also a driver of condensate formation.

According to our results, Sup35N is sufficient for condensate formation, whereas Sup35M antagonizes this process (Fig. 1), although this effect is only seen when Sup35M is fused to Sup35N, and not *in trans* (Fig. 1*H*). As Sup35M is dispensable for both condensate formation and dissolution, the cluster of charged residues that is located within the C-proximal portion of Sup35M and is implicated in the modulation of LLPS in response of acidic stress (48) is evidently not crucial for the regulation of condensate formation during osmotic stress. It is possible that Sup35M interferes with phase separation *via* repulsive interactions between charged residues (48) and/or *via* interactions with chaperones (64), Pub1 and microtubules (65), or other proteins. Sup35C may also interfere with condensate formation *via* interactions with functional components of translational machinery; however, in contrast to its postulated role during starvation-related acidic stress (49), this interaction is certainly not crucial during osmotic stress, as Sup35N/NM condensates are readily detectable in *sup35-ΔNM* cells, where Sup35C is physically separated from Sup35N/NM. Notably, Sup35N is a major driver of amyloid formation, partly antagonized by Sup35M and Sup35C (40, 66). This points to the parallels between roles of various Sup35 regions during formation of various types of supramolecular assemblies.

It remains to be seen whether Sup35-based condensates induced by osmotic stress include other proteins and/or nucleic acids. Formation of these condensates in [*pin*^*-*^ *psi*^*-*^] cells and their appearance as individual globular structures clearly distinguishes them from the previously reported “bead” assemblies composed of chaperones and Sup35 amyloid fibrils (62). However, the incorporation of some proteins and/or RNAs, as in yeast heat shock–induced stress granules (67) or acidic stress–induced puncta (49), cannot be ruled out and remains a topic for further investigation.

### Relationships between stress, liquid condensates, and amyloids

Osmotic stress has previously been implicated in promoting [*PSI*^*+*^] prion formation in the presence of [*PIN*^*+*^] prion by a destabilized derivative of Sup35 containing extra copies of oligopeptide repeats (35). In our hands, osmotic stress promoted [*PSI*^*+*^] formation in the presence of excess Sup35N-HA (but not Sup35N-YFP) in cells lacking other prions (Fig. 7*A*), suggesting that the attachment of a fluorophore somewhat antagonizes the prion nucleation potential.

Similar to LLPS induced by acidification (48), ability of Sup35NM to form condensates during hyperosmotic stress is conserved in yeast evolution (Fig. 6). In contrast to its *S. cerevisiae* counterpart, Sup35N/NM from a distantly related species, *O. methanolica*, promotes formation of both liquid condensates and filamentous amyloid assemblies in *S. cerevisiae* cells lacking any preexisting prions (Fig. 6). Notably, liquid condensates formed by *O. methanolica* Sup35 N/NM-YFP convert into filamentous amyloids in *S. cerevisiae* [*pin*^*-*^] cells (Figs. 9 and S4). Consistent with this observation, osmotic stress promotes [*PSI*^*+*^] formation in an *S. cerevisiae* strain lacking endogenous Sup35 and containing chimeric protein with the Sup35NM region from *O. methanolica*, both in the presence and in the absence of the *O. methanolica* Sup35N/NM-YFP construct (Fig. 8, *A*–*B*).

Some condensates of *S. cerevisiae* Sup35N-YFP (and more rarely, condensates of Sup35NM-YFP) convert into the 1,6-HD–resistant (possibly gel-like or semisolid) assemblies after prolonged incubation with osmotic stressors; however, in contrast to *O. methanolica* Sup35N/NM bearing constructs, formation of filamentous amyloids by these condensates was not detected (Figs. 4, *B* and 5, *A*). *In vitro*, formation of liquid Sup35NM condensates may either facilitate or inhibit amyloid nucleation, depending on concentration (50). Measurements of protein levels (Fig. S5) rule out the possibility of that increased amyloid formation by *O. methanolica* Sup35 derivatives is due to increased protein levels, as compared to *S. cerevisiae* Sup35 derivatives. The following mutually nonexclusive hypotheses could explain differences between *O. methanolica* and *S. cerevisiae* condensates.

1) Conversion of condensates of endogenous Sup35_Sc_ PrD into amyloids is counteracted by *S. cerevisiae* cellular components (possibly chaperones) that are not capable of counteracting amyloid formation by the heterologous *O. methanolica* protein with comparable efficiency.
2) The *O. methanolica* Sup35 protein is generally more prone to amyloid nucleation compared to other Sup35 orthologs tested in this study. Indeed, while the Sup35N regions of all these proteins are NQ-rich, *O. methanolica* Sup35N exhibits the highest ratio of N to Q residues among all these proteins (Fig. S6 and Table S31). Derivatives of yeast prion proteins (including Sup35) with increased N-content relative to Q-content have been shown to better propagate the prion state *in vivo* (68), and this pattern correlates with an increased ability of a protein to cross-seed other amyloids (69).

In general, our data shows that formation of liquid condensates by Sup35-derived constructs in yeast cells can be induced by conditions other than acidic stress (specifically, by hyperosmotic stress), and that, at least for *O. methanolica* Sup35, reversible liquid condensates represent intermediates in the formation of amyloid-based prions *in vivo*, rather than an alternative type of assembly. This model provides a unique opportunity for monitoring *in vivo* condensate-to-amyloid transition in real time by fluorescence microscopy.

### Possible biological roles of Sup35 condensates

It has been reported that Sup35 condensates formed during acidic stress facilitate recovery from stress (48), although this observation is challenged by other authors (49). Protective dynamic assemblies that are dependent on Pub1 (TIA-1) protein, which include some components of translational machinery and are termed stress granules, have been detected during both heat shock and glucose starvation (70, 71). However, formation of “classic” yeast stress granules has not been detected during some other stresses, including hyperosmotic or hypoosmotic stress (72, 73). It seems likely that Sup35 condensates (which may include other proteins) represent a functional analog of stress granules produced in different stressful conditions. In our hands, hyperosmotic stress is toxic to yeast cells from early exponential cultures, although little or no toxicity was detected in cells from late exponential cultures (Fig. 2*E*). Notably, condensate accumulation is increased in osmotically stressed late exponential or stationary cultures, compared to early exponential cultures (Fig. 2*D*). This suggests that Sup35-containing condensates are involved in protection from stress. Notably, condensate-containing cells tend to delay resumption of cell division after return to nonstress conditions, compared to cells without condensates (Fig. 2, *F*–*G*). This occurs despite condensates typically being dissolved at early stages of recovery and could be likened to the previously reported delay in translation and resumption of cell division until stress granules are dissolved (67, 74).

Notably, presence or absence of the prion form of the chimeric Sup35 protein with *O. methanolica* PrD did not significantly affect viability of *S. cerevisiae* cells during osmotic stress, although some differences were detected between different prion variants (Fig. 8*E*). On the other hand, it is known that many variants of the [*PSI*^+^] prion are detrimental to yeast cells at normal growth conditions (75). One intriguing possibility is that condensate formation may decrease a possibility of the stress-induced conversion of Sup35 into an amyloid that may become toxic upon resumption of growth. This protective mechanism may not work in case of a heterologous protein from *O. methanolica*, which readily converts from condensate to amyloid during or after osmotic stress in *S. cerevisiae* cells. Further research is needed to explore this scenario.

## Experimental procedures

### Strains and cultivation conditions

*S. cerevis*iae strains used in this study are listed in Table S32. Strains GT81-1C ([*PSI*^*+*^ *PIN*^*+*^]), GT159 ([*psi*^-^ *PIN*^+^]), GT409 ([*psi*^-^ *pin*^-^]), and GT1569 (*hsp104Δ*) were haploid isogenic derivatives of GT81 (56), while the [*psi*^-^ *PIN*^+^] strain GT12, containing the *sup35-ΔNM* allele was a derivative of 74-D694 (40). The previously described (76) [*PSI*^*+*^ *PIN*^*+*^] strain SY831, containing the mCherry-coding sequence inserted in frame between *SUP35N* and *SUP35M*, was constructed and kindly provided by T. Serio. The strain 1074 with GFP insertion between *SUP35N* and *SUP35M* regions (77) was constructed and kindly provided by D. Masison. The [*psi*^-^ *pin*^-^] strains AB190, GT2508, and GT2518 were derivatives of GT12, SY831, and 1074, respectively, cured of prions by GuHCl. The [*psi*^-^ *pin*^-^] strain GT225-6B of GT81 origin contained *sup35Δ* deletion on the chromosome, and the chimeric gene *SUP35(NM)*_*Om*_*-C*_*Sc*_, composed of *SUP35NM* of *O. methanolica* and *SUP35C* of *S. cerevisiae* and expressed from the endogenous *S. cerevisiae SUP35* promoter (*P*_*SUP35*_) on the low-copy (*CEN*) plasmid (56). All strains contained the *ade1-14* (UGA) nonsense reporter allele, used for the phenotypic detection of nonsense-suppression by [*PSI*^+^] (26). Standard yeast media and cultivation conditions were used (78). Rich organic medium (YPD) contained 1% yeast extract, 2% peptone, and 2% dextrose; standard synthetic medium contained 0.67% yeast nitrogen base without amino acids or ammonium sulfate, 0.5% ammonium sulfate, 2% dextrose, and 13 nutrition supplements (adenine, arginine, histidine, isoleucine, leucine, lysine, methionine, phenylalanine, threonine, tryptophan, tyrosine, uracil, and valine) unless some were specifically dropped as indicated (*e.g.,* −Ura for the medium lacking uracil). Solid media contained 1.5% agar (US Biologicals). Yeast cultures were grown at 30 °C unless specified otherwise. Standard yeast synthetic media contains 3 μM Cu^++^, allowing for background expression from the *P*_*CUP1*_ promoter, while overexpression was typically induced by addition of 100 μM CuSO_4_, which is not toxic for most laboratory *S. cerevisiae* yeast strains (79, 80), including those employed in our experiments. For cultures used in FM, an additional 200 mg/l of adenine was added to the incubation medium in order to suppress the accumulation of red pigment. Presence and stringency of [*PSI*^*+*^] prion were monitored by growth on synthetic medium lacking adenine (-Ade), and by intensity of red color on the complete medium with decreased yeast extract concentration (1/4 YPD) as previously described (26). To cure yeast prions by GuHCl, cultures were incubated on solid YPD with 5 mM GuHCl for three consecutive passages (about 40 generations), followed by streaking out on YPD and analyzing individual colonies. GuHCl inhibits the Hsp104 chaperone, thus blocking fragmentation and proliferation of prion polymers (81, 82). *S. cerevisiae*–*Esherichia coli* shuttle vectors were propagated in the standard *E. coli* strains XL1-blue, XL10-gold, or DH5α.

### Plasmids

*S. cerevisiae*–*E. coli CEN* (low-copy) shuttle vectors bearing either *URA3* or *LEU2* marker were used in this study (Table S33). Primers used in plasmid constructions are shown in Table S34. Original vectors pRS315 (*LEU2*) and pRS316 (*URA3*) were kindly provided by P. Hieter (83). Centromeric *URA3* plasmid pmCUP1 bearing the copper-inducible *P*_*CUP1*_ promoter was kindly provided by S. Lindquist (84). The multicopy plasmid p426MET25_sfpHluorin (MRV55), RRID:Addgene_115697, kindly provided by E. Boles (51) and bearing the sfpHluorin gene under the control of the methionine-repressible promoter *P*_*MET25*_, was employed for measuring cytosolic pH.

Plasmids bearing *SUP35NM* regions from *S. paradoxus* (*Sp*) or *S. uvarum* (*Su*) fused to YFP and placed under the control of *P*_*CUP1*_ were produced by inserting *Bam*HI-*Sac*II *SUP35NM* fragments from pmCUP1-Sup35NMSp-sGFP or pmCUP-Sup35NMSbay-sGFP plasmids (58), respectively, into pmCUP1-Sup35NM-YFP, kindly provided by S. Lindquist (85) and cut by the same enzymes, thus replacing *SUP35NM* of *S. cerevisiae*. The Sup35NM-coding region of *N. castellii* (*Nc*) was amplified by PCR (with the addition of *Bam*HI and *Sac*II restriction sites) from the genomic DNA of *N. castellii* strain FM476, kindly provided by M. Johnston, digested by *Bam*HI and *Sac*II, and ligated into pmCUP1-Sup35NM_Sc_-YFP, cut by the same enzymes, thus producing the plasmid pmCUP1-Sup35NM_Nc_-YFP (*LEU2*). The plasmid pmCUP1-LEU2 was created by inserting the *P*_*CUP*1_ promotor from the pmCUP1 (*URA3*) plasmid into the pRS315 vector using *Eco*RI and *Bam*HI restriction sites. The pmCUP1-YFP (*LEU2*) plasmid was produced by inserting the YFP-coding region, PCR-amplified from pmCUP1-Sup35NM_Sc_-YFP, with the addition of *Not*I and *Sac*I restriction sites, into pmCUP1-LEU2, digested with *Not*I and *Sac*I. To create the pmCUP1-Sup35(NM)_Om_-YFP (*LEU2*) plasmid, *SUP35NM* of *O. methanolica* (*Om*) was amplified from genomic DNA of an *O. methanolica* strain kindly provided by I. Tostorukov, with the addition of *Bam*HI and *Xba*I restriction sites, digested, and ligated into pmCUP1-YFP plasmid cut by the same enzymes. To construct the pmCUP1-Sup35N_Om_-YFP (*LEU2*) plasmid, the *SUP35(NM)*_*Om*_ fragment of pmCUP1-Sup35(NM)_Om_-YFP plasmid was cut off with *Bam*HI and *Xba*I and replaced with the *SUP35N*_*Om*_ region, PCR-amplified from pmCUP1-Sup35(NM)_Om_-YFP with the addition of the same restriction sites. The plasmid p316CUP1-Sup35NM_Sc_-YFP (*URA3*) was constructed by inserting the *Bam*HI-*Sac*I *S. cerevisiae SUP35NM-YFP* fragment from pmCUP1-Sup35NMSc-YFP (*LEU2*) into the pmCup1 plasmid, cut at the same restriction sites. The plasmid p316CUP1-Sup35N_Sc_-YFP (*URA3*) was produced by replacing the *Bam*HI-*Xb*aI *S. cerevisiae SUP35NM* fragment of p316CUP1-Sup35NM_Sc_-YFP plasmid by the *S. cerevisiae SUP35N* region, PCR-amplified from p316CUP1-Sup35NMSc-YFP with the addition of *Bam*HI and *Xba*I sites.

### Osmotic stress experiments

For the treatment with osmotic stressors, yeast cells were grown for 24 h with shaking from starting *A*_600_ = 0.2 in YPD, or in synthetic medium selective for the plasmid for strains containing Sup35N/NM encoding plasmids, pelleted, resuspended in H_2_O with or without a respective stressor, incubated for specified periods of time, precipitated by 3 to 5 min centrifugation at 3000*g*, and either used for FM or washed in H_2_O and plated onto respective media as described in the text.

### Analysis of [PSI^+^] formation

To detect [*PSI*^+^] formation, either decimal serial dilutions of yeast cultures were spotted onto YPD and -Ade, or onto complete and adenine-lacking plasmid-selective media for spot plate assays, or calculated numbers of cells were plated onto respective media for quantitative assays. Plates were scored after three (YPD, -Ura, and -Leu) or 14 days (-Ade, -Ura-Ade, or -Leu-Ade) at 30 °C. The frequency of [*PSI*^+^] prion formation was calculated as the ratio of the number of colonies grown on the medium without adenine to the number of colonies grown on respective complete medium, accounting for dilution. At least four independent biological repeats were performed in each case. To confirm that Ade^+^ colonies contain the [*PSI*^+^] prion, curability of Ade^+^ phenotype by GuHCl was checked, as described above.

To detect [*PSI*^*+*^] prion formation in response to osmotic stress, overnight cultures were diluted to *A*_600_ = 0.2 in 5 ml of appropriate media (either YPD, or plasmid-selective media with 100 μM CuSO_4_ for the overproducer strains). After 24 h of growth, four 1-ml aliquots of each culture were collected, with one of them used as pretreatment control, while cells from each of other three aliquots were precipitated at 3000*g* for 3 min, washed with 1 ml of H_2_O, and resuspended each in 1 ml of either H_2_O, 1 M, or 2 M KCl, followed by 24 h of incubation, collecting cells by centrifugation, washing in 1 ml of H_2_O, and either spotting decimal serial dilutions or plating calculated numbers of cells onto the complete and –Ade media. Viabilities during stress were calculated as ratios between colonies grown on complete medium before and after stress.

### FM analysis

FM was performed using BX41 (Olympus) microscope with 100×/NA 1.5 oil objective and Olympus DP-71 color digital camera, Leica DM6000B microscope with 100×/1.30 oil ph3 (UplanFl) objective, or (in case of higher resolution) confocal Leica TCS SP5 (Leica Microsystems GmbH) microscope. FRAP measurements employed the Leica Stellaris 5 system with a 63×/1.40 oil (HC PL APO CS2) objective, with 512 × 512 pixels image resolution, 488 nm excitation wavelength, 490 to 750 nm emission detection window, 0.001 line time, and line average of 1. Microscopy slides were pretreated with 1 mg/ml concanavalin A (Sigma-Aldrich). For each sample, 420 frames were collected with a time interval of 0.518 s, including 20 frames collected prior to high laser power point bleaching, and 400 frames collected immediately after bleaching. Single normalization to the prebleach signal in the region of interest was performed as described (86).

For treatment with 1,6-HD, cells were incubated for 10 min at room temperature in H_2_O, containing 10 μg/ml digitonin (or 0.1% of Triton X-100), 10% 1,6-HD (Ferak), and a respective stressor in the case of osmotic stress, and examined by FM.

For staining intracellular assemblies with ThT, 1 ml of yeast cells culture were first washed in PBS at pH 7.4, then stained for 20 min in a 30 μM ThT solution in PBS containing 0.1% Triton X-100, and washed five times in PBS. ThT fluorescence was detected using CFP filter set (Olympus). The 3D reconstructions and videos were created using FIJI software (RRID:SCR_002285; https://imagej.net/software/fiji/) (87).

For time-lapse microscopy, yeast cells were immobilized on a microscope slide using poly-L-lysine (20 μl of a 1% solution poured and dried) or 1.2% agarose pads prepared in the corresponding medium or 1 M KCl. After applying the cells to the prepared slide, the sample was covered with a coverslip. The edges of the coverslip along the long side of the slide were sealed either with double-sided tape or nontoxic modeling clay. To change conditions, the new solution was added from one side of the sample, while absorption was performed from the other side using Whatman paper. Images were captured by BX41 (Olympus) microscope every 20 min for up to 5 h, or short 2-min videos were created, depending on the purpose of the experiment.

### Protein analysis

For SDS-PAGE, yeast cells were grown for 24 h with shaking from starting *A*_600_ = 0.2 in 5 to 10 ml of synthetic medium, selective for the plasmid, and precipitated at 3000*g* for 5 min from 1.5 ml of the culture. Proteins were isolated as described (88) with modifications, and analyzed by SDS-PAGE and Western blotting, followed by reaction to respective antibodies. Cell pellets were treated with 300 μl of 2M LiAc, 0.4 M NaOH on ice for 5 min, resuspended in 100 μl of SDS-PAGE sample buffer (60 mM Tris–HCl pH 6.8, 2% SDS, 10% glycerol, 2% 2-mercaptoethanol, and 0.002% bromophenol blue), and boiled for 5 min. Then, supernatants cleared of cell debris by centrifugation were run on SDS-polyacrylamide (8–12%, depending on the size of protein of interest) gel with 4% stacking gel in Tris–glycine–SDS running buffer (25 mM Tris, 192 mM glycine, 0.1% SDS, pH 8.3), followed by electrotransfer to a PVDF or nitrocellulose membrane (Thermo Fisher Scientific, 0.45 μm), preblocking with 5% nonfat milk or 2% Amersham ECL Prime Blocking Reagent (GE Healthcare), probing with the appropriate antibody and visualization with Amersham ECL Detection Reagents (Cytiva). The following antibodies were employed: rabbit anti-GFP (Evrogen Cat# AB011, RRID:AB_2892560), or anti-Rnq1, kindly provided by Dr S. Lindquist, with secondary enhanced chemiluminescence (ECL) horseradish peroxidase (HRP)-linked anti-rabbit donkey IgG (Cytiva Cat# NA934, RRID:AB_772206); rabbit anti-Sup35С kindly provided by D. Bedwell, with secondary HRP-linked anti-rabbit goat IgG (Sigma-Aldrich Cat# A6154, RRID:AB_258284); rabbit anti-GFP, also recognizing YFP (Sigma-Aldrich Cat# G1544, RRID:AB_439690) and rabbit anti-G6PDH (Sigma-Aldrich Cat# A9521, RRID:AB_258454) kindly provided by M. Torres with secondary HRP-linked anti-rabbit goat IgG (Sigma-Aldrich Cat# A6154, RRID:AB_258284).

In case of gel entry assay, which assesses the ability of proteins to enter a polyacrylamide gel, extracts were split into two aliquots, with one aliquot boiled for 5 min and the other remaining unboiled, and analyzed by SDS-PAGE and Western blotting as described above.

SDD-AGE was performed as described previously (89, 90) with modifications. Proteins were isolated from yeast cells, grown for 24 h with shaking from starting *A*_600_ = 0.2 in 50 ml of synthetic medium selective for the respective plasmid, precipitated at 1600*g* for 10 min, washed with and resuspended in 300 μl of cold lysis buffer (50 mM Tris–HCl pH 7.5, 150 mM NaCl, 10 mM EDTA, 10 mM DTT, 10 mM PMSF, and 1*X* protease inhibitor cocktail from Sigma P8215), and disrupted by agitation with 300 μl glass beads (Sigma-Aldrich) for 10 min at 4 °C. Cell debris was spun down at 1600 g for 10 min at 4 °C, and supernatants, transferred to a new tube, were stored at −80 °C until use. Protein concentrations were determined by Bradford assay (Bio-Rad). About 20 μl of each extract were incubated with the equal amount of 2*X* loading buffer (80 mM Tris, 40 mM acetate, 2 mM EDTA, 20% glycerol, 2% SDS, 0.06% bromophenol blue, and 100 mM DTT) either at room temperature for 7 min (nonboiled samples), or in a boiling water bath for 7 min (boiled samples), and run on 1.8% agarose gel containing 0.1% SDS in tris-acetate-EDTA buffer (40 mM Tris, 20 mM acetate, and 1 mM EDTA) at 110 V at 4 °C, followed by transfer to the nitrocellulose membrane *via* capillary blotting with TBS buffer (50 mM Tris, 10 mM NaCl, pH 7.5) and reaction to respective antibodies and detection using ECL chemiluminescent reagent (GE Healthcare). The abovementioned Sup35C or rabbit anti-GFP primary antibodies (Evrogen Cat# AB011, RRID:AB_2892560) with secondary ECL anti-rabbit HRP-linked donkey IgG (Cytiva Cat# NA934, RRID:AB_772206) were used for detection.

### Protein level comparisons

To measure protein levels using densitometry, SDS-PAGE and Western blotting were performed as described above, followed by densitometry using ImageJ (https://imagej.net/ij/) software (NIH). The intensity of each protein band was normalized to the corresponding loading control (G6PDH or full-length Sup35, where appropriate). Data were expressed as a ratio of the target protein to the loading control, and results were averaged from at least three (typically more) independent experiments. Final data were normalized to the amount of Sup35NM_Sc_-YFP without overproduction, with this amount set to 1. If more than one replicate of Sup35NM_Sc_-YFP was present on the gel, data were normalized to the average level of Sup35NM_Sc_-YFP.

To measure levels of YFP or YFP-tagged proteins by fluorescence intensity, cells expressing respective constructs were grown for 24 h with shaking in 5 to 10 ml of appropriate synthetic medium, selective for the a respective plasmid, with starting *A*_600_ of 0.2, precipitated from 1 ml of culture by centrifugation for 5 min at 3000*g*, and resuspended in 1 ml PBS containing 137 mM NaCl, 2.7 mM KCl, 8 mM Na_2_HPO_4_, and 2 mM KH_2_PO_4_, to *A*_600_ of 6.0. Fluorescence was measured in 200 μl samples using 96-well microtitre plates, on SpectraMax iD3-2053 (Molecular Devices), with shaking before measurements, at 505 nm excitation and 545 nm emission wavelengths. Measurements were normalized to absorbance, also recorded on the same plate reader.

### Measurements of cytosolic pH

The measurements of cytosolic pH were carried out as described (51). The pH-sensitive fluorescent protein, sfpHluorin was expressed from the plasmid p426MET25_sfpHluorin (MRV55) at high levels under control of the methionine repressible promoter *P*_*MET25*_, in the absence of methionine. Yeast cells were cultured in 5 ml of low fluorescent synthetic medium (synthetic medium without riboflavin and folic acid) lacking uracil and methionine. After overnight growth, the culture was diluted to *A*_600_ = 0.1 and grown for additional 4 h. To draw a calibration curve, yeast cells were harvested by centrifugation at 3000*g* for 3 min, incubated in 2.5 ml of PBS (137 mM NaCl, 2.7 mM KCl, 8 mM Na_2_HPO_4_, and 2 mM KH_2_PO_4_) containing 100 μg/ml of digitonin for 30 min at 30 °C to permeabilize membranes, washed in 5 ml of PBS, resuspended in PBS at *A*_600_ = 20, and diluted to *A*_600_ = 0.3 in 200 μl of citric acid/Na_2_HPO_4_ (McIlvaine) buffer with pH ranging within 5.0 to 8.0 in increments of 0.5. Measurements of fluorescence were performed on a Sparc 10M microplate spectrofluorometer from TECAN, after 20 min of incubation at room temperature. Fluorescence of sfpHluorin was measured at an emission wavelength of 512 nm with different excitation wavelengths, ranging in 5-nm increments from 355 nm to 475 nm (Fig. 3*B*). The excitation spectrum of sfpHluorin is pH-dependent, with the maximum fluorescence intensity at 385 nm for high pH and at 465 nm for low pH; thus, each pH is characterized by the respective ratio of fluorescence intensities at 385 nm and 465 nm (*R*_*385/465*_). A calibration curve was plotted for the emission fluorescence intensity at 512 nm as a function of the 385 nm/465 nm ratio and pH of the buffer (Fig. 3*C*). Each measurement was taken in four biological replicates. The autofluorescence of yeast cells was taken into account by subtracting the fluorescence intensity of sfpHluorin-free cells at 385 and 465 nm from corresponding intensities of cells expressing sfpHluorin. The formula for calculating corrected *R*_*385/465*_ is as follows:

$$R_{385/465}=\frac{I_{pH\left(385\right)}-I_{a\left(385\right)}}{I_{pH\left(465\right)}-I_{a\left(465\right)}\;}$$

where *I*_*pH*_ is the fluorescence intensity of cells overproducing sfpHluorine at indicated excitation wavelength (385 or 465 nm), and *I*_*a*_ is the autofluorescence of cells at corresponding excitation wavelength.

To measure the impact of hyperosmotic stress on intracellular pH, yeast cells were harvested by centrifugation at 3000*g* for 3 min and resuspended in low fluorescent medium lacking folic acid and riboflavin at final *A*_600_ = 20, followed by a dilution in aqueous solutions with various osmotic stressors (1 M KCl, 1 M NaCl, 1.5 M or 2.7 M sorbitol, or 2 М glycerol) to *A*_600_ = 0.3. The fluorescence emission intensity of the sfpHluorin protein was measured at an emission wavelength of 512 nm with excitation wavelengths of 385 nm and 465 nm within 30 min after the start of hyperosmotic stress. Next, R385/465 was calculated (accounting for cell autofluorescence), and pH was determined using the calibration curve, composed as described above, Fig. 3, *A*–*B*. Each measurement was performed in four biological replicates.

To measure the impact of intracellular pH on YFP fluorescence, yeast cells overproducing Sup35N-YFP (in the presence of 100 μM CuSO_4_) were harvested by centrifugation at 3000*g* for 3 min, incubated in 2.5 ml of PBS containing 100 μg/ml digitonin for 30 min at 30 °C to permeabilize membranes, washed in 5 ml of PBS, resuspended in PBS at *A*_600_ = 20, and diluted to *A*_600_ = 0.3 in 200 μl of citric acid/Na_2_HPO_4_ (McIlvaine) buffer with pH ranging from 3.0 to 8.0 in increments of 1. Measurements of pH were performed on a Spark 10 M microplate spectrofluorometer from TECAN, after 20 min of incubation at room temperature. Fluorescence of YFP was measured at an emission wavelength of 535 nm and an excitation wavelength of 490 nm.

### Statistical comparisons

Error bars represent either SDs, calculated according to a standard formula (91), or in case of some FM data, standard errors of proportion, calculated as follows: Standard error = √(*p(1−p)/n*), where *p* is the frequency of a given class and *n* is the total number of cells in sample. Statistical significance of differences was determined using either Fisher’s exact test for some FM data or Student’s *t* test for other experiments. Differences with *p* ≤ 0.05 were considered significant. On figures, *p* values are indicated by stars as follows: ∗ ≤0.05, ∗∗≤0.01, and ∗∗∗≤0.001.

To assess the effect of osmotic stress using FM, paired experiments were conducted with measurements taken before and after treatment within the same clone. Since the analysis was performed in actively dividing cultures, variability between different cultures occurred in some cases, but changes were consistently observed in one direction, confirming statistical significance. In these instances, one representative replicate is shown on a Figure. For most important comparisons, all replicates are shown in Supporting information tables.

## Data availability

Data are available in the article itself and its supplementary materials. Original data, strains, and plasmids generated in this study are accessible from the corresponding author upon request.

## Supporting information

This paper contains supporting information including references (51, 56, 58, 59, 80, 83, 85, 87, 92, 93).

## Conflict of interest

The authors declare that they have no conflicts of interest with the contents of this article.

## References

[1] Feng Z., Jia B., Zhang M. (2021). Liquid-liquid phase separation in biology: specific stoichiometric molecular interactions vs promiscuous interactions Mediated by Disordered Sequences. Biochemistry.

[2] Antifeeva I.A., Fonin A.V., Fefilova A.S., Stepanenko O.V., Povarova O.I., Silonov S.A. (2022). Liquid-liquid phase separation as an organizing principle of intracellular space: overview of the evolution of the cell compartmentalization concept. Cell. Mol. Life. Sci..

[3] Evreinova T.N., Mamontova T.W., Karnauhov V.N., Stephanov S.B., Hrust U.R. (1974). Coacervate systems and origin of life. Orig. Life.

[4] McSwiggen D.T., Mir M., Darzacq X., Tjian R. (2019). Evaluating phase separation in live cells: diagnosis, caveats, and functional consequences. Genes. Dev..

[5] Zhou X., Sumrow L., Tashiro K., Sutherland L., Liu D., Qin T. (2022). Mutations linked to neurological disease enhance self-association of low-complexity protein sequences. Science.

[6] Franzmann T.M., Alberti S. (2019). Protein phase separation as a stress survival strategy cold spring. Harb. Perspect. Biol..

[7] Yoshizawa T., Nozawa R.S., Jia T.Z., Saio T., Mori E. (2020). Biological phase separation: cell biology meets. Biophys. Rev..

[8] Alberti S., Dormann D. (2019). Liquid-liquid phase separation in disease. Annu. Rev. Genet..

[9] Gao C., Gu J., Zhang H., Jiang K., Tang L., Liu R. (2022). Hyperosmotic-stress-induced liquid-liquid phase separation of ALS-related proteins in the nucleus. Cell Rep..

[10] Coskuner-Weber O., Mirzanli O., Uversky V.N. (2022). Intrinsically disordered proteins and proteins with intrinsically disordered regions in Neurodegenerative Diseases. Biophys. Rev..

[11] Watts J.C., Prusiner S.B. (2018). Beta-amyloid prions and the pathobiology of Alzheimer's disease. Cold Spring Harb. Perspect. Med..

[12] Prusiner S.B. (2013). Biology and genetics of prions causing neurodegeneration. Annu. Rev. Genet..

[13] Shao J., Diamond M.I. (2007). Polyglutamine diseases: emerging concepts in pathogenesis and therapy. Hum. Mol. Genet..

[14] Uversky V.N. (2017). Looking at the recent advances in understanding alpha-synuclein and its aggregation through the proteoform prism. F1000Res.

[15] Jucker M., Walker L.C. (2018). Propagation and spread of pathogenic protein assemblies in neurodegenerative diseases. Nat. Neurosci..

[16] Wilson C.J., Bommarius A.S., Champion J.A., Chernoff Y.O., Lynn D.G., Paravastu A.K. (2018). Biomolecular assemblies: moving from observation to predictive design. Chem. Rev..

[17] Otzen D., Riek R. (2019). Functional amyloids. Cold Spring Harb. Perspect. Biol..

[18] Rubel M.S., Fedotov S.A., Grizel A.V., Sopova J.V., Malikova O.A., Chernoff Y.O. (2020). Functional mammalian amyloids and amyloid-like Proteins. Life (Basel).

[19] Chen C., Ding X., Akram N., Xue S., Luo S.Z. (2019). Fused in sarcoma: properties, self-assembly and correlation with Neurodegenerative Diseases. Molecules.

[20] Ray S., Singh N., Kumar R., Patel K., Pandey S., Datta D. (2020). alpha-Synuclein aggregation nucleates through liquid-liquid phase separation. Nat. Chem..

[21] Murray D.T., Kato M., Lin Y., Thurber K.R., Hung I., McKnight S.L. (2017). Structure of FUS protein fibrils and its relevance to self-assembly and phase separation of low-complexity domains. Cell.

[22] Babinchak W.M., Haider R., Dumm B.K., Sarkar P., Surewicz K., Choi J.K. (2019). The role of liquid-liquid phase separation in aggregation of the TDP-43 low-complexity domain. J. Biol. Chem..

[23] Kanaan N.M., Hamel C., Grabinski T., Combs B. (2020). Liquid-liquid phase separation induces pathogenic tau conformations in vitro. Nat. Commun..

[24] Ambadipudi S., Biernat J., Riedel D., Mandelkow E., Zweckstetter M. (2017). Liquid-liquid phase separation of the microtubule-binding repeats of the Alzheimer-related protein Tau. Nat. Commun..

[25] Petronilho E.C., Pedrote M.M., Marques M.A., Passos Y.M., Mota M.F., Jakobus B. (2021). Phase separation of p53 precedes aggregation and is affected by oncogenic mutations and ligands. Chem. Sci..

[26] Liebman S.W., Chernoff Y.O. (2012). Prions in yeast. Genetics.

[27] Wickner R.B. (2016). Yeast Fungal Prions. Cold Spring Harb. Perspect. Biol..

[28] Roberts B.T., Wickner R.B. (2003). Heritable activity: a prion that propagates by covalent autoactivation. Genes Dev..

[29] Harvey Z.H., Chakravarty A.K., Futia R.A., Jarosz D.F. (2020). A prion epigenetic switch establishes an active chromatin state. Cell.

[30] Itakura A.K., Chakravarty A.K., Jakobson C.M., Jarosz D.F. (2020). Widespread prion-based control of growth and differentiation strategies in Saccharomyces cerevisiae. Mol. Cell.

[31] Wickner R.B., Edskes H.K., Kryndushkin D., McGlinchey R., Bateman D., Kelly A. (2011). Prion diseases of yeast: amyloid structure and biology. Semin. Cel. Dev. Biol..

[32] Saupe S.J., Jarosz D.F., True H.L. (2016). Amyloid Prions in Fungi. Microbiol. Spectr..

[33] Halfmann R., Jarosz D.F., Jones S.K., Chang A., Lancaster A.K., Lindquist S. (2012). Prions are a common mechanism for phenotypic inheritance in wild yeasts. Nature.

[34] Wickner R.B. (2019). Anti-prion systems in yeast. J. Biol. Chem..

[35] Tyedmers J., Madariaga M.L., Lindquist S. (2008). Prion switching in response to environmental stress. PLoS Biol..

[36] Caudron F., Barral Y. (2013). A super-assembly of Whi3 encodes memory of deceptive encounters by single cells during yeast courtship. Cell.

[37] Chernova T.A., Kiktev D.A., Romanyuk A.V., Shanks J.R., Laur O., Ali M. (2017). Yeast short-lived actin-associated protein forms a metastable prion in response to thermal. Stress Cell Rep..

[38] Chernoff Y.O., Grizel A.V., Rubel A.A., Zelinsky A.A., Chandramowlishwaran P., Chernova T.A. (2020). Application of yeast to studying amyloid and prion diseases. Adv. Genet..

[39] Chernoff Y.O., Derkach I.L., Inge-Vechtomov S.G. (1993). Multicopy SUP35 gene induces de-novo appearance of psi-like factors in the yeast Saccharomyces cerevisiae. Curr. Genet..

[40] Derkatch I.L., Chernoff Y.O., Kushnirov V.V., Inge-Vechtomov S.G., Liebman S.W. (1996). Genesis and variability of [PSI] prion factors in Saccharomyces cerevisiae. Genetics.

[41] Derkatch I.L., Bradley M.E., Zhou P., Chernoff Y.O., Liebman S.W. (1997). Genetic and environmental factors affecting the de novo appearance of the [PSI+] prion in Saccharomyces cerevisiae. Genetics.

[42] Derkatch I.L., Bradley M.E., Hong J.Y., Liebman S.W. (2001). Prions affect the appearance of other prions: the story of [PIN(+)]. Cell.

[43] Osherovich L.Z., Weissman J.S. (2001). Multiple Gln/Asn-rich prion domains confer susceptibility to induction of the yeast [PSI(+)] prion. Cell.

[44] Chernoff Y.O., Uptain S.M., Lindquist S.L. (2002). Analysis of prion factors in Yeast. Methods Enzymol..

[45] Zhou P., Derkatch I.L., Liebman S.W. (2001). The relationship between visible intracellular aggregates that appear after overexpression of Sup35 and the yeast prion-like elements [PSI(+)] and [PIN(+)]. Mol. Microbiol..

[46] Ganusova E.E., Ozolins L.N., Bhagat S., Newnam G.P., Wegrzyn R.D., Sherman M.Y. (2006). Modulation of prion formation, aggregation, and toxicity by the actin cytoskeleton in yeast. Mol. Cell Biol..

[47] Dorweiler J.E., Lyke D.R., Lemoine N.P., Guereca S., Buchholz H.E., Legan E.R. (2022). Implications of the actin cytoskeleton on the multi-step process of [PSI(+)] prion formation. Viruses.

[48] Franzmann T.M., Jahnel M., Pozniakovsky A., Mahamid J., Holehouse A.S., Nuske E. (2018). Phase separation of a yeast prion protein promotes cellular fitness. Science.

[49] Grimes B., Jacob W., Liberman A.R., Kim N., Zhao X., Masison D.C. (2023). The properties and domain requirements for phase separation of the Sup35 prion protein *in vivo*. Biomolecules.

[50] Fukuyama M., Nishinami S., Maruyama Y., Ozawa T., Tomita S., Ohhashi Y. (2023). Detection of fibril nucleation in micrometer-sized protein condensates and suppression of Sup35NM fibril nucleation by liquid-liquid phase separation. Anal. Chem..

[51] Reifenrath M., Boles E. (2018). A superfolder variant of pH-sensitive pHluorin for in vivo pH measurements in the endoplasmic reticulum. Sci. Rep..

[52] Griesbeck O., Baird G.S., Campbell R.E., Zacharias D.A., Tsien R.Y. (2001). Reducing the environmental sensitivity of yellow fluorescent protein. Mechanism applications. J. Biol. Chem..

[53] Alberti S., Saha S., Woodruff J.B., Franzmann T.M., Wang J., Hyman A.A. (2018). A user’s guide for phase separation assays with purified proteins. J. Mol. Biol..

[54] Mitrea D.M., Chandra B., Ferrolino M.C., Gibbs E.B., Tolbert M., White M.R. (2018). Methods for physical characterization of phase-separated bodies and membrane-less organelles. J. Mol. Biol..

[55] Chernoff Y.O., Lindquist S.L., Ono B., Inge-Vechtomov S.G., Liebman S.W. (1995). Role of the chaperone protein Hsp104 in propagation of the yeast prion-like factor [psi+]. Science.

[56] Chernoff Y.O., Galkin A.P., Lewitin E., Chernova T.A., Newnam G.P., Belenkiy S.M. (2000). Evolutionary conservation of prion-forming abilities of the yeast Sup35 protein. Mol. Microbiol..

[57] Jensen M.A., True H.L., Chernoff Y.O., Lindquist S. (2001). Molecular population genetics and evolution of a prion-like protein in Saccharomyces cerevisiae. Genetics.

[58] Chen B., Newnam G.P., Chernoff Y.O. (2007). Prion species barrier between the closely related yeast proteins is detected despite coaggregation. Proc. Natl. Acad. Sci. U. S. A..

[59] Chandramowlishwaran P., Sun M., Casey K.L., Romanyuk A.V., Grizel A.V., Sopova J.V. (2018). Mammalian amyloidogenic proteins promote prion nucleation in yeast. J. Biol. Chem..

[60] Ferreira P.C., Ness F., Edwards S.R., Cox B.S., Tuite M.F. (2001). The elimination of the yeast [PSI+] prion by guanidine hydrochloride is the result of Hsp104 inactivation. Mol. Microbiol..

[61] Jung G., Jones G., Masison D.C. (2002). Amino acid residue 184 of yeast Hsp104 chaperone is critical for prion-curing by guanidine, prion propagation, and thermotolerance. Proc. Natl. Acad. Sci. U. S. A..

[62] Alexandrov A.I., Grosfeld E.V., Dergalev A.A., Kushnirov V.V., Chuprov-Netochin R.N., Tyurin-Kuzmin P.A. (2019). Analysis of novel hyperosmotic shock response suggests 'beads in liquid' cytosol. Structure Biol. Open.

[63] Khan T., Kandola T.S., Wu J., Venkatesan S., Ketter E., Lange J.J. (2019). Quantifying nucleation in vivo reveals the physical basis of prion-like phase behavior. Mol. Cell.

[64] Helsen C.W., Glover J.R. (2012). Insight into molecular basis of curing of [PSI+] prion by overexpression of 104-kDa heat shock protein (Hsp104). J. Biol. Chem..

[65] Li X., Rayman J.B., Kandel E.R., Derkatch I.L. (2014). Functional role of Tia1/Pub1 and Sup35 prion domains: directing protein synthesis machinery to the tubulin cytoskeleton. Mol. Cell.

[66] Kochneva-Pervukhova N.V., Poznyakovski A.I., Smirnov V.N., Ter-Avanesyan M.D. (1998). C-terminal truncation of the Sup35 protein increases the frequency of de novo generation of a prion-based [PSI+] determinant in Saccharomyces cerevisiae. Curr. Genet..

[67] Grousl T., Ivanov P., Malcova I., Pompach P., Frydlova I., Slaba R. (2013). Heat shock-induced accumulation of translation elongation and termination factors precedes assembly of stress granules in S. cerevisiae. PLoS one.

[68] Halfmann R., Alberti S., Krishnan R., Lyle N., O’Donnell C.W., King O.D. (2011). Opposing effects of glutamine and asparagine govern prion formation by intrinsically disordered proteins. Mol. Cell.

[69] Shattuck J.E., Waechter A.C., Ross E.D. (2017). The effects of glutamine/asparagine content on aggregation and heterologous prion induction by yeast prion-like domains. Prion.

[70] Buchan J.R., Parker R. (2009). Eukaryotic stress granules: the ins and outs of translation molecular. Cell.

[71] Cherkasov V., Grousl T., Theer P., Vainshtein Y., Glasser C., Mongis C. (2015). Systemic control of protein synthesis through sequestration of translation and ribosome biogenesis factors during severe heat stress. FEBS Lett..

[72] Buchan J.R., Muhlrad D., Parker R. (2008). P bodies promote stress granule assembly in Saccharomyces cerevisiae. J. Cell Biol..

[73] Kilchert C., Weidner J., Prescianotto-Baschong C., Spang A. (2010). Defects in the secretory pathway and high Ca2+ induce multiple P-bodies. Mol. Biol. Cell.

[74] Cherkasov V., Hofmann S., Druffel-Augustin S., Mogk A., Tyedmers J., Stoecklin G. (2013). Coordination of translational control and protein homeostasis during severe heat. Stress Curr. Biol..

[75] McGlinchey R.P., Kryndushkin D., Wickner R.B. (2011). Suicidal [PSI+] is a lethal yeast prion. Proc. Natl. Acad. Sci. U. S. A..

[76] Vishveshwara N., Bradley M.E., Liebman S.W. (2009). Sequestration of essential proteins causes prion associated toxicity in yeast. Mol. Microbiol..

[77] Song Y., Wu Y.X., Jung G., Tutar Y., Eisenberg E., Greene L.E. (2005). Role for Hsp70 chaperone in Saccharomyces cerevisiae prion seed replication. Eukaryot. Cell.

[78] Sherman F. (2002). Getting started with yeast. Methods Enzymol..

[79] Etcheverry T. (1990). Induced expression using yeast copper metallothionein promoter. Methods Enzymol..

[80] Serio T.R., Cashikar A.G., Moslehi J.J., Kowal A.S., Lindquist S.L. (1999). Yeast prion [psi +] and its determinant, Sup35p. Methods Enzymol..

[81] Jung G., Masison D.C. (2001). Guanidine hydrochloride inhibits Hsp104 activity in vivo: a possible explanation for its effect in curing yeast prions. Curr. Microbiol..

[82] Eaglestone S.S., Ruddock L.W., Cox B.S., Tuite M.F. (2000). Guanidine hydrochloride blocks a critical step in the propagation of the prion-like determinant [PSI(+)] of Saccharomyces cerevisiae. Proc. Natl. Acad. Sci. U. S. A..

[83] Sikorski R.S., Hieter P. (1989). A system of shuttle vectors and yeast host strains designed for efficient manipulation of DNA in Saccharomyces cerevisiae. Genetics.

[84] Patino M.M., Liu J.J., Glover J.R., Lindquist S. (1996). Support for the prion hypothesis for inheritance of a phenotypic trait in yeast. Science.

[85] Derkatch I.L., Uptain S.M., Outeiro T.F., Krishnan R., Lindquist S.L., Liebman S.W. (2004). Effects of Q/N-rich, polyQ, and non-polyQ amyloids on the de novo formation of the [PSI+] prion in yeast and aggregation of Sup35 *in vitro*. Proc. Natl. Acad. Sci. U. S. A..

[86] Phair R.D., Scaffidi P., Elbi C., Vecerova J., Dey A., Ozato K. (2004). Global nature of dynamic protein-chromatin interactions in vivo: three-dimensional genome scanning and dynamic interaction networks of chromatin proteins. Mol. Cell Biol..

[87] Schindelin J., Arganda-Carreras I., Frise E., Kaynig V., Longair M., Pietzsch T. (2012). Fiji: an open-source platform for biological-image analysis. Nat. Methods.

[88] Zhang T., Lei J., Yang H., Xu K., Wang R., Zhang Z. (2011). An improved method for whole protein extraction from yeast Saccharomyces cerevisiae. Yeast.

[89] Howie R.L., Jay-Garcia L.M., Kiktev D.A., Faber Q.L., Murphy M., Rees K.A. (2019). Role of the cell asymmetry apparatus and ribosome-associated chaperones in the destabilization of a Saccharomyces cerevisiae prion by heat shock. Genetics.

[90] Kushnirov V.V., Alexandrov I.M., Mitkevich O.V., Shkundina I.S., Ter-Avanesyan M.D. (2006). Purification and analysis of prion and amyloid aggregates. Methods.

[91] MacDonald J.H. (2009).

[92] Kushnirov V.V., Kochneva-Pervukhova N.V., Chechenova M.B., Frolova N.S., Ter-Avanesyan M.D. (2000). Prion properties of the Sup35 protein of yeast Pichia methanolica. EMBO J..

[93] Notredame C., Higgins D.G., Heringa J. (2000). T-Coffee: a novel method for fast and accurate multiple sequence alignment. J. Mol. Biol..

