# Supplementary material for: Osmotic stress induces formation of both liquid condensates and amyloids by a yeast prion domain
Source: J Biol Chem. 2024 Sep 12;300(10):107766. doi: 10.1016/j.jbc.2024.107766 (PMC11736011; doi:10.1016/j.jbc.2024.107766)
Supplement: Supporting information [file mmc3.pdf]

Supporting Information for

Osmotic stress induces formation of both liquid condensates and amyloids by a yeast prion domain

Anastasia V. Grizel, Natalia A. Gorsheneva, Jonathan B. Stevenson, Jeremy Pflaum, Florian Wilfling, Aleksandr A. Rubel, and Yury O. Chernoff

Corresponding author: Yury O. Chernoff, School of Biological Sciences, Georgia Institute of Technology, Krone EBB, 950 Atlantic Drive NW, Atlanta, Georgia 30332-2000, USA

Email: yury.chernoff@biology.gatech.edu

This file includes:

Figures S1 to S6 – pages S-2 to S-7

Tables S1 to S34 – pages S-8 to S-27

Video Descriptions S1 to S9 – pages S-28 to S-30

Dataset Descriptions S1 and S2 – page S-31

Other supporting materials for this manuscript include the following:

Videos S1 to S9

Datasets S1 and S2

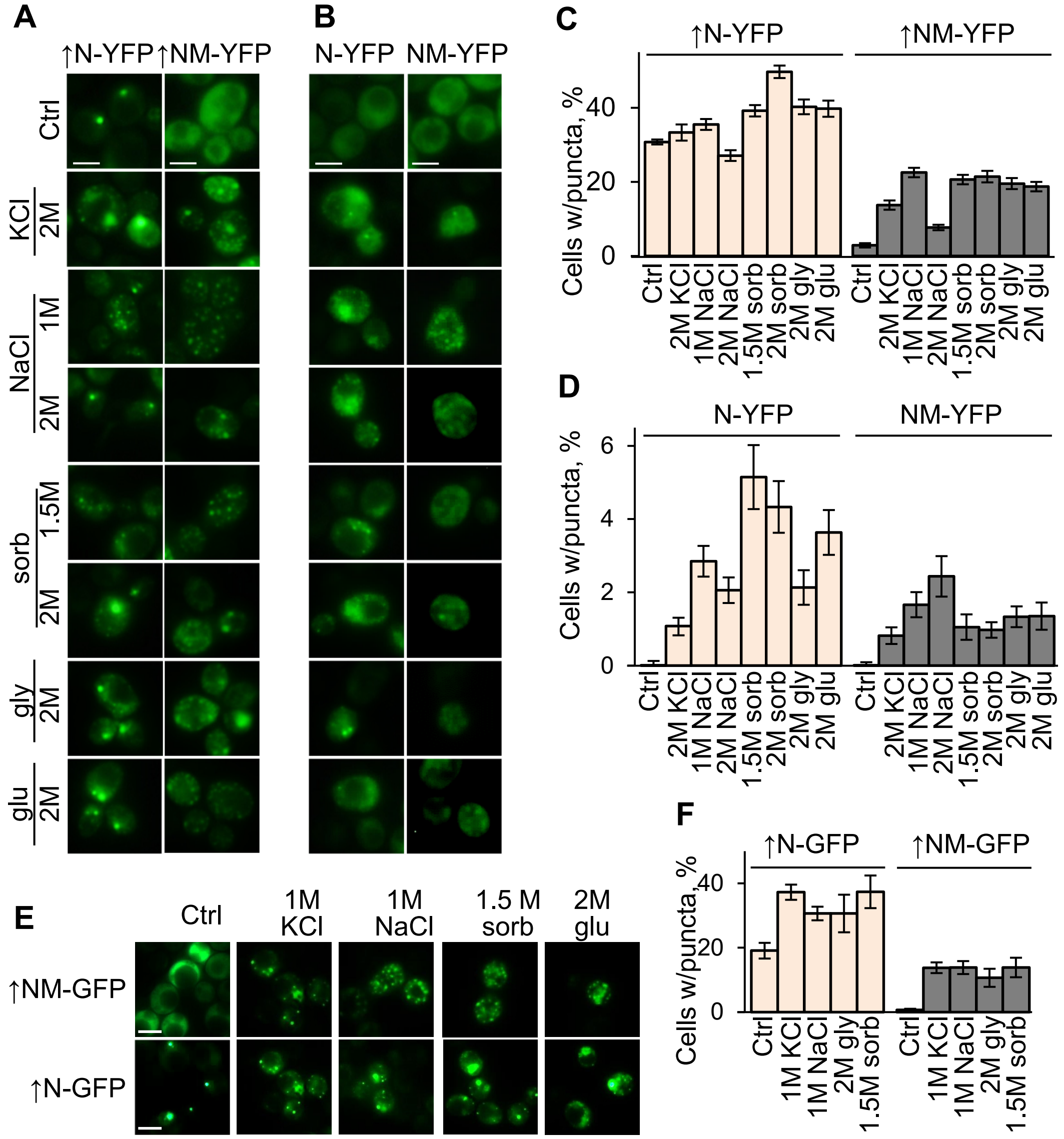

Figure S1. Detection of supramolecular assemblies of fluorophore-tagged Sup35 constructs upon overproduction and/or after osmotic stress. Fluorescent micrographs depict the assemblies of Sup35N and Sup35NM (tagged with YFP or GFP as shown) in [*psi*$^-$ *pin*$^-$] *sup35-ΔNM* cells after 5 min in various osmotic agents, as indicated; sorb, gly and glu refer to sorbitol, glycerol and glucose respectively. Fluorophore-tagged constructs were either induced at high levels by addition of 100 μM $CuSO_4$ (A, C, E, F), or produced at moderate levels at background concentrations, 3 μM of Cu^{++} (B, D). "Ctrl" refers to non-stressed cultures. Panels A, B, and E show representative fluorescent images, while panels C, D, and F show proportions of cells with puncta among all cells exhibiting fluorescence. Error bars correspond to SEs. Scale bars correspond to 5 μm. The control (Ctrl) cells in panels A and B, and control numbers in panels C and D are identical to control cells shown in Figs. 1A (*ΔNM*) and 1D, and to control numbers shown in Figs. 1B and 1E, respectively, as they originate from one and the same set of experiments in each case.

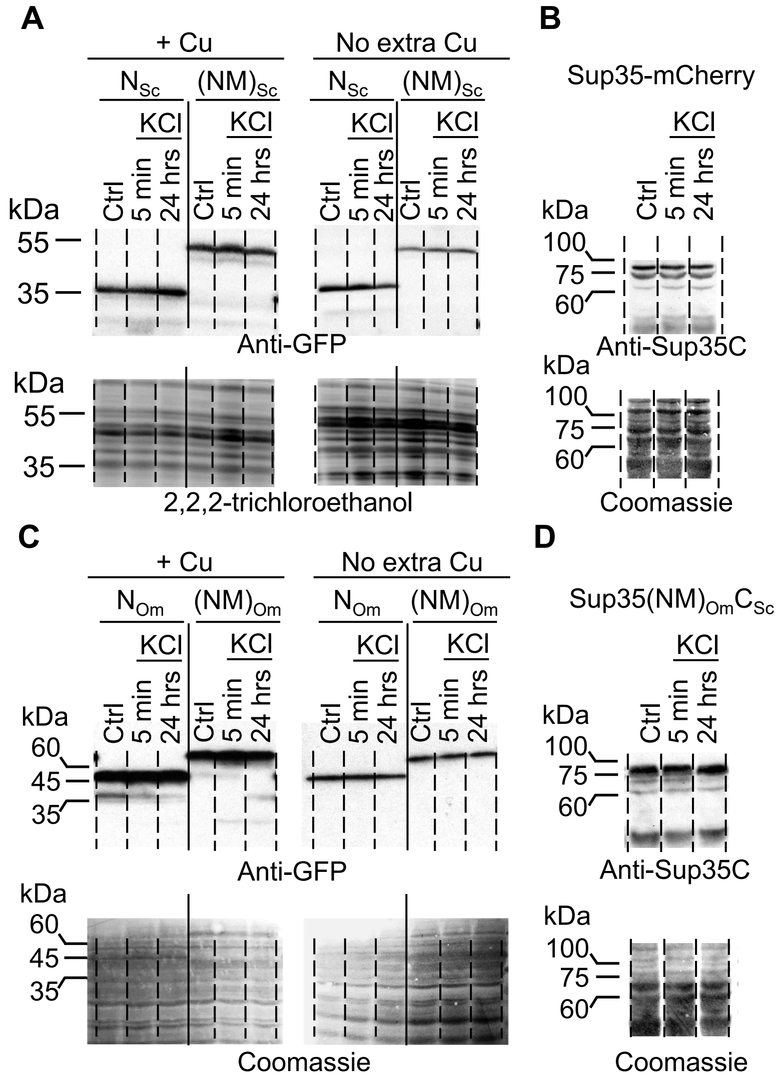

Figure S2. Abundance of Sup35 and Sup35-derived constructs with and without osmotic stress. Proteins were isolated from control cells grown in the cultural medium (Ctrl), and from cells after short (5 min) or long (24 hrs) exposure to the osmotic stressor (1M KCl). Results of Western blotting followed by reaction to respective antibodies (as indicated), along with the respective control PVDF membranes stained with 2,2,2-trichlorethanol or Coomassie (as indicated) for total protein are shown. (A) Levels of Sup35N-YFP, N_{Sc} or Sup35NM-YFP, $(NM)_{Sc}$ [*pin*⁻ *psi*⁻] *sup35-ΔNM* cultures grown in synthetic media either containing background levels, 3 μM Cu^{++} (No extra Cu) or with additional 100 μM $CuSO_4$ (+ Cu). (B) Levels of Sup35-mCherry protein produced from the chromosomal gene under the endogenous P_{SUP35} promoter. (C) Levels of Sup35N_{Om}-YFP, N_{Om} or Sup35$(NM)_{Om}$-YFP, $(NM)_{Om}$ in [*psi*⁻ *pin*⁻] *sup35-ΔNM* cells also containing a chimeric Sup35$(NM)_{Om}$-C_{Sc} protein and grown in synthetic media either with background or increased levels of Cu^{++}. (D) Levels of chimeric Sup35$(NM)_{Om}$-C_{Sc} protein produced from a construct under the endogenous promotor in [*pin*⁻ *psi*⁻] *sup35-ΔNM* cells.

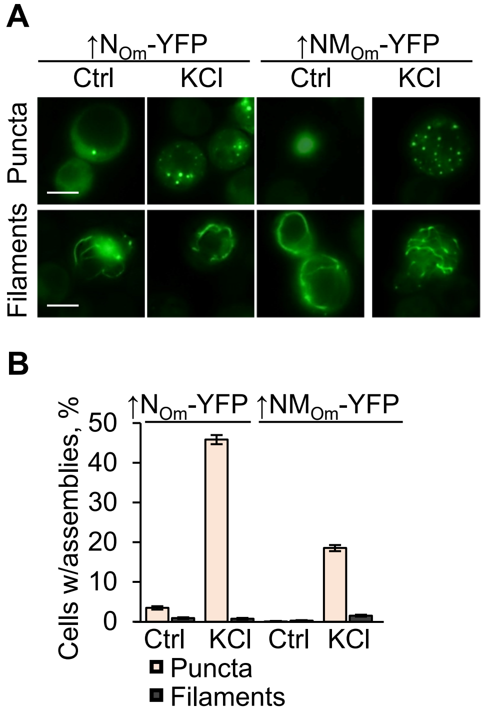

Figure S3. Protein assemblies formed by *O. methanolica* Sup3N/NM-YFP proteins in the [*psi*$^-$ *pin*$^-$] strain containing the chimeric Sup35NM$_{Om}$-C$_{Sc}$ protein. (A, image) and (B, percentages) - Formation of different types of protein assemblies without stress (Ctrl) and after 5-min incubation with 1M KCl. Scale bars correspond to 5 µm.

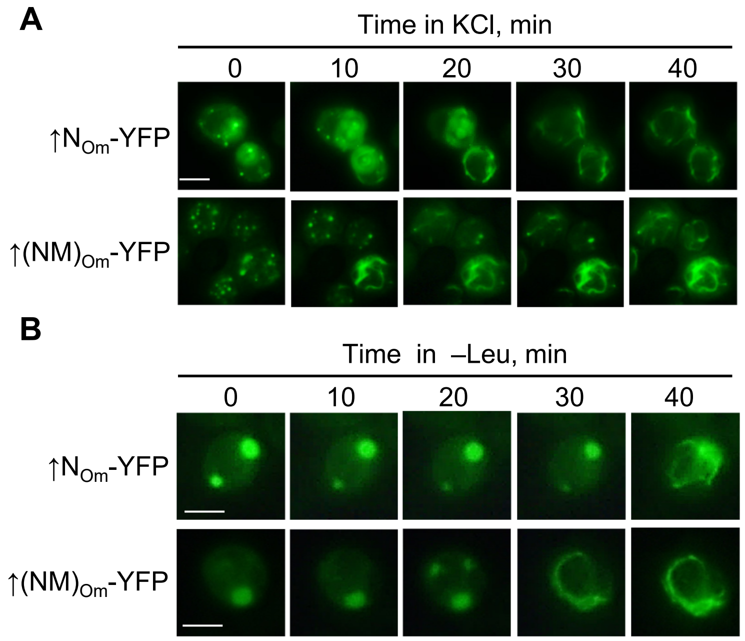

Figure S4. Conversion of *O. methanolica* Sup35N/NM-YFP condensates to amyloids in strain with chimeric Sup35(NM)$_{Om}$-C$_{Sc}$ protein. Time-lapse monitoring of the conversion of condensates formed by overproduced (after addition of 100 μM $CuSO_4$) *O. methanolica* Sup35N/NM-YFP into the filamentous amyloid assemblies during stress in the [*psi*$^-$ *pin*$^-$] strain with chimeric Sup35(NM)$_{Om}$-C$_{Sc}$ protein (A), or without stress in the [*psi*$^-$ *pin*$^-$] strain with *sup35-ΔNM* deletion (B). Conversion was detected in 22 out of 28 (N$_{Om}$-YFP, panel A), 22 out of 26 [(NM)$_{Om}$-YFP, panel A], 1 out of 3 (N$_{Om}$-YFP, panel B) and 1 out of 5 [(NM)$_{Om}$-YFP, panel B] cells with condensates tested. Scale bars correspond to 5 μm.

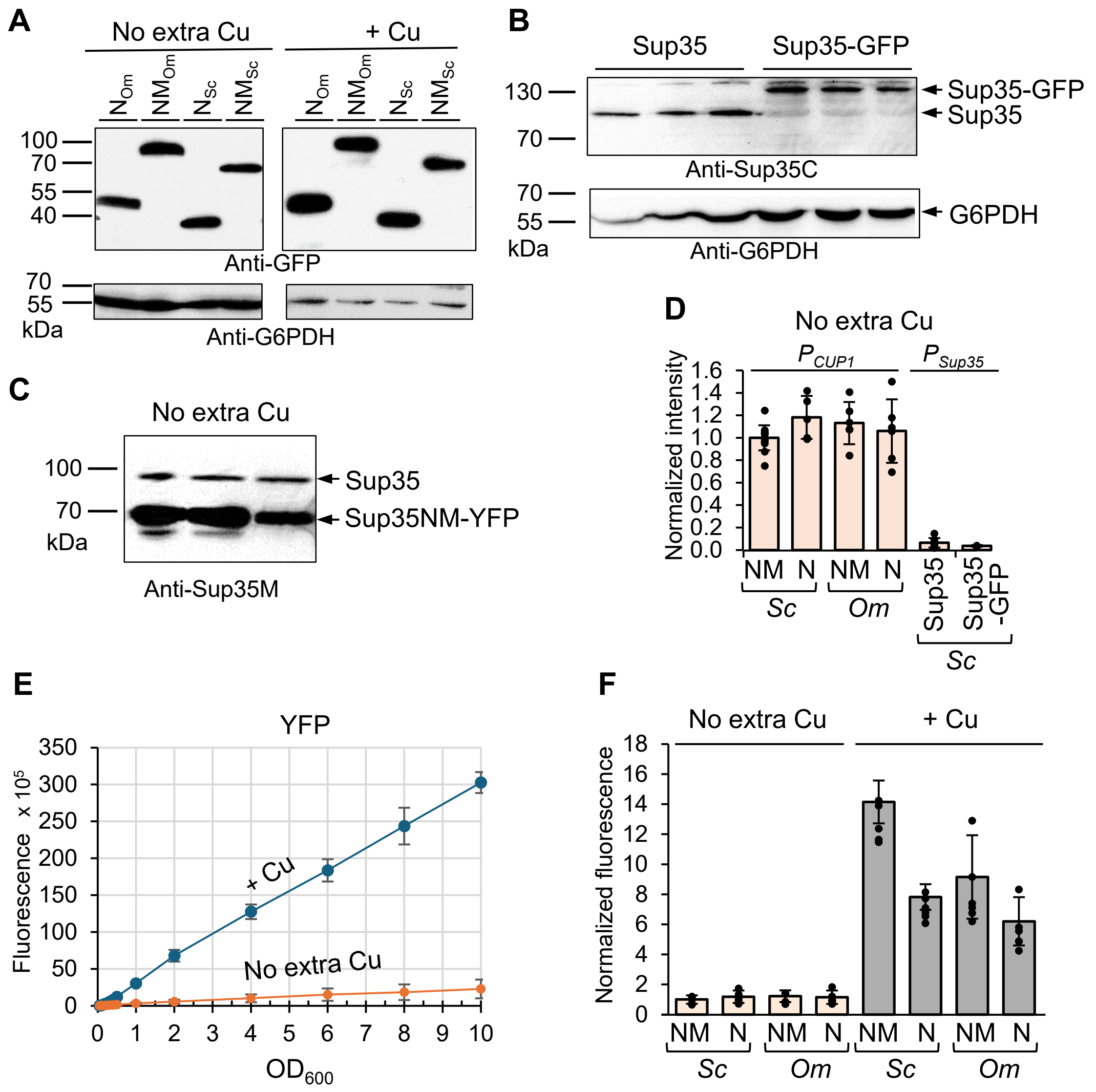

Figure S5. Comparison of protein levels for Sup35 and various Sup35-derived constructs. (A) Representative Western blot results for Sup35N/NM-YFP of *S. cerevisiae* (*Sc*) and *O. methanolica* (*Om*) expressed in the *S. cerevisiae sup35-ΔNM* strain AB190 under the inducible P_{CUP1} promoter. Cells were grown in synthetic media, either containing background levels, 3 μM of Cu^{++} (No extra Cu) or with additional 100 μM $CuSO_4$ (+ Cu). Chimeric proteins were detected by anti-GFP antibody, with G6PDH used as a loading control. (B) Western blot comparing the levels of endogenous Sup35 and Sup35-GFP proteins produced by chromosomal genes under the endogenous P_{SUP35} promoter in the strains GT17 and GT2518, respectively. Proteins were detected by anti-Sup35C antibody, with G6PDH used as a loading control. (C) Western blot comparing the levels of endogenous Sup35 with levels of Sup35NM-YFP produced from P_{CUP1} promoter at background levels of Cu^{++}. Proteins were detected by anti-Sup35M antibody, with G6PDH used as a loading control. (D) Comparison of normalized levels for proteins shown on panels (A), (B) and (C) using densitometry, in the conditions without extra Cu^{++}; in each case, levels are shown in relation to Sup35(NM)$_{Sc}$-YFP expressed in respective conditions (E) Linear dependence of fluorescence signal from YFP-producing cells on cell concentration, measured by absorbance of the culture at 600 nm (A_{600}) in the cells of *S. cerevisiae sup35-ΔNM* strain AB190 bearing a plasmid with the YFP gene under the P_{CUP1} promoter. Cultures were grown for 24 hrs with or without extra 100 μM $CuSO_4$ as indicated, then serial dilutions were performed, and fluorescence was measured using a microplate reader at 545 nm. Linear relationship is detected at least within the A_{600} range from 2 to 8. (F) Comparison of the levels of *Sc* and *Om* Sup35N/NM-YFP proteins at background and increased levels of Cu^{++}, in relation Sup35(NM)$_{Sc}$-YFP, as measured by cell fluorescence.

```
Sc     1 MSDSNQGNNQ-----------------------------------  10
Om     1 MSQDQQQQQQFNANNLAGNVQNINLNAPAYDPAVQSYIPNTAQAF  45
cons   1 **:.:* ::*                                     45

Sc    11 ----------QNYQQYSQNGNQQQGNNRYQGYQAYNAQAQPAGGY  45
Om    46 VPSAQPYIPGQQEQQFGQYGQQQQNYNQ-GGYNNYNNR----GGY  85
cons  46           *: **:.* *:***. *:  **: ** :    ***  90

Sc    46 YQNYQGYSGYQQGGYQQYNPDAGYQQQYNPQGGYQQYNPQGGYQQ  90
Om    86 SNNRGGYNNSNRGGYSNYNSY---NTN-SNQGGYSNYNNNYANNS 126
cons  91  :*  **.. ::***.:**.    : : . ****.:** : . :. 135

Sc    91 QFNPQGGRGNYKNFNYNNNL-QGY----------QAGFQPQSQGM 124
Om   127 YN---------NNNNYNNNYNQGYNNYNSQPQGQDQQQETGSGQM 162
cons 136            :* *****  ***          :   :. *  * 180

Sc   125 SLNDFQKQQKQAA----PKPKKTLKL-VSSSGIKLANATKKVGTK 164
Om   163 SLEDYQKQQKESLNKLNTKPKKVLKLNLNSSTVKAPIVTKKKEEE 207
cons 181 **:*:*****::     .****.*** :.** :* . .***   : 225

Sc   165 PAESDKKEEEKSAE---TKEPTKEPTKVEEP-----------VKK 195
Om   208 PVNQESKTEEPAKEEIKNQEPAEAENKVEEESKVEAPTAAKPVSE 252
cons 226 *.:.:.* ** : *   .:**::  .****            *.: 270

Sc   196 EEKPVQTEEKTEEKS--ELPKVEDLKISESTHNTNNANVTSADAL 238
Om   253 SEFPASTP-KTEAKASKEVAAAAAALKKEVSQAKKESNVTNADAL 296
cons 271 .* *..*  *** *:  *:. .     .* :: .:::***.**** 315

Sc   239 IKEQEEEVDDEVVND 253
Om   297 VKEQEEQIDASI--- 308
cons 316 :*****::* .:    330
```

Figure S6. Amino acid sequence alignments of *S. cerevisiae* and *O. methanolica* Sup35NM regions. *Sc* and *Om* indicate *S. cerevisiae* (UniProt database, P05453) and *O. methanolica* (92) amino acid sequences, respectively. "Cons" corresponds to the consensus sequence; designations "*", ":", and "." indicate identical amino acid residues, residues of similar properties, and semi-conserved substitutions, respectively. The N domain is displayed in bold font. Sequences were aligned using the T-coffee multiple sequence alignment package (93).

Supplementary Tables

Table S1. Effect of 1M KCl on condensate formation by Sup35N/NM-YFP, overproduced in the presence of 100 µM $CuSO_4$ in *sup35-ΔNM* and WT strains. (Data for Fig. 1B).

Strain	Protein	Culture	Before stress			1M KCl		
			With condensates		Total*	With condensates		Total*
			Number	%		Number	%	
AB190 [*psi*$^-$ *pin*$^-$] *sup35-ΔNM*	Sup35N-YFP	**1**	**195**	**30.8 +/-1.8**	**634**	**543**	**51.7 +/-1.5**	**1050**
		2	31	3.7	833	270	24.3	1113
		3	18	2.8	640	216	26.3	821
		4	39	5.9	661	240	19.6	1227
		5	135	15.4	878	107	26.4	406
		6	85	14.1	605	127	25.4	500
		7	86	23.2	371	98	36.7	267
		8	22	4.2	526	45	12.1	371
		9	23	4.5	513	114	14.0	817
		10	119	15.1	789	411	33.6	1225
		11	58	11.0	527	123	28.3	434
		12	119	9.3	1282	107	38.6	277
		13	29	4.9	588	166	27.2	610
		14	136	24.9	547	340	48.6	700
	Sup35NM-YFP	**1**	**23**	**2.9 +/-0.6**	**796**	**276**	**21.4 +/-1.1**	**1292**
		2	4	0.7	602	67	5.0	1339
		3	3	0.2	1303	91	6.7	1359
		4	6	0.5	1274	39	5.5	708
		5	11	1.3	848	75	7.8	963
		6	13	4.8	271	100	25.7	389
		7	5	1.6	317	20	3.9	512
		8	2	1.3	158	63	11.0	574
		9	21	4.7	448	64	11.5	558
		10	6	0.6	1028	92	10.1	908
		11	7	0.4	1621	146	6.9	2134
		12	7	0.4	1616	125	6.6	1904
		13	3	0.7	408	50	4.5	1123
		14	8	1.5	543	30	4.5	666
		15	18	2.76	651	52	10.5	495
GT409 [*psi*$^-$ *pin*$^-$] WT	Sup35N-YFP	**1**	**117**	**20.4 +/-1.7**	**573**	**65**	**56.5 +/-4.6**	**115**
		2	26	5.9	439	291	41.3	704
		3	8	24.2	33	126	45.8	275
	Sup35NM-YFP	**1**	**13**	**2.9 +/-0.6**	**620**	**39**	**25.7+/ -3.5**	**152**
		2	19	9.0	211	140	34.7	403
		3	12	5.2	232	28	18.2	154

*Total number of cells with fluorescence (the same designation is used in Tables S2 through S8, S14 through S19, and S27, below).
For each strain/plasmid combination, cultures # 1 (in bold) are presented on Fig. 1B; standard errors of proportion are shown (with +/-) for these cultures.

Table S2. Effect of various osmotic stresses on condensate formation by Sup35N/NM-YFP, overproduced in the presence of 100 μM $CuSO_4$ in the [*psi⁻ pin⁻*] *sup35-ΔNM* strain. (Data for Fig. S1C.)

Protein	Stressor	Cells with condensates			Total
		Number	%	SE*, %	
Sup35N-YFP	No stress*	195	30.8	1.8	634
	2M KCl	155	33.3	2.2	465
	1M NaCl	379	35.5	1.5	1067
	2M NaCl	261	27.1	1.4	963
	1.5M sorbitol	398	39.2	1.5	1015
	2M sorbitol	434	49.7	1.7	873
	2M glycerol	244	40.3	2.0	606
	2M glucose	202	39.8	2.2	508
Sup35NM-YFP	No stress*	23	2.9	0.6	796
	2M KCl	105	13.7	1.2	764
	1M NaCl	243	22.5	1.3	1078
	2M NaCl	100	7.7	0.7	1300
	1.5M sorbitol	208	20.6	1.3	1008
	2M sorbitol	150	21.4	1.6	700
	2M glycerol	137	19.5	1.5	701
	2M glucose	176	18.7	1.3	939
YFP***	No stress	0	0	0	5295
	1M KCl	0	0	0	3455

* Data for non-stressed cultures are repeated from Table S1 (cultures #1), because the same cultures were used in this experiment.
** Standard error of proportion (the same designation is used in Tables S3 through S8, S14 through S19, and S27, below).
***Cumulative data for 6 independent cultures.

Table S3. Effect of osmotic stress on number of condensates per cell by Sup35N/NM-YFP, overproduced in the presence of 100 μM $CuSO_4$. (Data for Fig. 1C.)

Protein	Stressor	Cells with > 4 condensates			Total
		Number	%	SE*, %	
Sup35N-YFP	No stress	0	0	0.01	5244
	1M KCl	282	6.2	0.5	2544
Sup35NM-YFP	No stress	1	0.01	0.01	8169
	1M KCl	652	25.6	0.6	4550

Table S4. Effect of various osmotic stressors on condensate formation by Sup35N/NM-GFP overproduced in the presence of 100 μM $CuSO_4$. (Data for Fig. S1F.)

Protein	Stressor	Cells with condensates			Total
		Number	%	SE, %	
Sup35NM-GFP	No stress	1	0.2	0.2	595
	1M KCl	58	13.8	1.7	421
	1M NaCl	41	13.9	2.0	296
	2M glycerol	13	10.7	2.8	122
	1.5M sorbitol	18	13.8	3.0	130
Sup35N-GFP	No stress	50	19.1	2.4	262
	1M KCl	155	37.3	2.4	416
	1M NaCl	148	30.6	2.1	483
	2M glycerol	19	30.6	5.9	62
	1.5M sorbitol	34	37.4	5.1	91

Table S5. Effect of 1M KCl on condensate formation by Sup35N/NM-YFP, expressed at moderate levels. (Data for Figs. 1E.)

Protein	Before stress				1M KCl		
	With condensates			Total	With condensates		Total
	Culture	Number	%		Number	%	
Sup35N-YFP	**1**	**0**	**0.0+/-0.1**	**830**	**51**	**2.6+/-0.4**	**1949**
	2	5	0.7	773	88	14.3	616
	3	0	0	659	26	2.0	1330
	4	3	0.4	824	35	7.7	453
	5	0	0	538	111	13.9	796
	6	0	0	461	46	7.4	618
Sup35NM-YFP	**1**	**0**	**0.0+/-0.1**	**1199**	**23**	**1.4+/-0.3**	**1649**

For each strain/plasmid combination, cultures # 1 (in bold) are presented on Fig. 1E; standard errors of proportion are shown (with +/-) for these cultures.

Table S6. Effect of various osmotic stressors on condensate formation by Sup35N/NM-YFP, expressed at moderate levels. (Data for Figs. S1D.)

Protein	Stressor	Cells with condensates			Total
		Number	%	SE, %	
Sup35N-YFP	No stress*	0	0.0	0.1	830
	2M KCl	18	1.1	0.3	1663
	1M NaCl	45	2.9	0.4	1579
	2M NaCl	34	2.1	0.4	1652
	1.5M sorbitol	33	5.2	0.9	640
	2M sorbitol	36	4.3	0.7	831
	2M glycerol	20	2.1	0.5	937
	2M glucose	34	3.6	0.6	935
Sup35NM-YFP	No stress*	0	0.0	0.1	1199
	2M KCl	13	0.8	0.2	1589
	1M NaCl	23	1.7	0.3	1383
	2M NaCl	19	2.4	0.6	779
	1.5M sorbitol	9	1.1	0.4	855
	2M sorbitol	21	1.0	0.2	2152
	2M glycerol	22	1.3	0.3	1648
	2M glucose	13	1.4	0.4	961

* Data for non-stressed cultures are repeated from Table S1 (cultures #1), because the same cultures were used in this experiment.

Table S7. Dissolution of Sup35NM-YFP condensates after removal of the osmotic stressor. (Data for Fig. 1F.)

Conditions	Culture	Cells with condensates			Total
		Number	%	SE, %	
Before stress (-Ura)	**1**	**25**	**2.1**	**0.4**	**1200**
	2	48	6.1	0.9	788
	3	115	18.5	1.6	622
Osmotic stress (1M KCl, 5-15 min)	**1**	**228**	**17.4**	**1.0**	**1310**
	2	438	28.8	1.2	1520
	3	603	41.8	1.3	1444
After stress (-Ura, 10 min)	**1**	**14**	**1.4**	**0.4**	**1020**
	2	20	3.5	0.8	570
	3	75	9.5	1.0	792

For each strain/plasmid combination, cultures # 1 (in bold) are presented on Fig. 1F.

Table S8. Effect of osmotic stress on condensate formation by full-length Sup35-mCherry protein expressed from chromosomal gene. (Data for Fig. 2B.)

Conditions	Cells with condensates			Total
	Number	%	SE, %	
No stress	16	1.3	0.3	1222
1M KCl	91	4.6	0.5	1981

Table S9. Effect of osmotic stress on condensate formation by full-length Sup35-GFP protein expressed from chromosomal gene. (Data for Fig. 2D.)

Conditions	Culture	Cells with condensates				Total
		Number	%	Mean	SD	
	1	1	0.1			873
Before stress	2	0	0.0			695
Late culture	3	0	0.0	0.4	0.5	389
(SC)	4	7	0.9			822
	5	7	1.0			681
Osmotic stress						
Late culture	1	18	1.0			1771
(1M KCl, 30	2	15	1.4	1.4	0.4	1108
min)	3	18	1.7			1088
Osmotic stress	1	329	33.3			987
Late culture	2	284	32.1	30.3	4.7	886
(1M KCl, 24	3	254	32.4			783
hrs)	4	178	23.2			766
Control	1	20	3.4			588
Late culture	2	11	2.5			444
(H_2O, 24 hrs)	3	44	6.6	3.4	1.9	667
	4	16	2.5			625
	5	13	1.9			686
Recovery*	1					
Late culture	2	22	2.8	2.9	0.2	776
(SC, 10 min)	3	14	2.8			498
		12	3.2			376
Osmotic stress,						
Early culture	6	76	8.5			893
(1M KCl, 24	7	88	9.4	8.9	0.5	938
hrs)	8	48	8.9			542
Before stress,	6	1	0.2			656
Early culture	7	1	0.1	0.2	0.6	820
(SC)	8	1	0.2			494

"Early" and "late" refer to early exponential (6 hrs of incubation) and late exponential or early stationary (24 hrs of incubation) growth phases, respectively.

* In synthetic medium after KCl treatment.

**Standard deviation (the same designation is used in Tables S10, S12, S13, S20, S22, S23, S28, S29 and S31, below).

Table S10. Resumption of budding by cells lacking or containing Sup35-GFP condensates after osmotic stress. (Data for Fig. 2G.)

Trial	Number of cells						Percent of cells with buds among cell types			
	With buds			Total			Diffused		With puncta	
	Diffused	With puncta	Total	Diffused	With puncta	Total	%	Mean ± SD	%	Mean ± SD
1	21	1	22	129	46	175	16.3		2.2	
2	11	13	24	41	85	126	26.8	22.2 ± 5.4	15.3	9.2 ± 6.6
3	15	5	20	64	49	113	23.4		10.2	

Table S11. Calibration of pH for *S. cerevisiae* cultures producing sfpHluorin. (Data for Fig. 3B.)

Culture	Buffer pH	$I_{pH(385)}$	$I_{pH(465)}$	$I_{a(385)}$	$I_{a(465)}$	$R_{385/465}$
#1	5.0	1051	2208	398.3	238.8	0.33
	5.5	1315	2786	401.8	231.0	0.36
	6.0	1712	3126	372.5	221.5	0.46
	6.6	2248	2719	353.3	231.0	0.76
	7.1	2667	2194	353.5	227.8	1.18
	7.7	3229	2052	471.0	233.3	1.52
	8.2	3595	1958	327.8	233.0	1.89
#2	5.0	802	1673	398.3	238.8	0.28
	5.5	1099	2492	401.8	231.0	0.31
	6.0	1254	2369	372.5	221.5	0.41
	6.6	1534	1949	353.3	231.0	0.69
	7.1	1729	1464	353.5	227.8	1.11
	7.7	2197	1351	471.0	233.3	1.54
	8.2	2390	1293	327.8	233.0	1.95
#3	5.0	1560	3734	398.3	238.8	0.33
	5.5	1652	3993	401.8	231.0	0.33
	6.0	2216	4312	372.5	221.5	0.45
	6.6	3345	4335	353.3	231.0	0.73
	7.1	3401	2812	353.5	227.8	1.18
	7.7	4912	2878	471.0	233.3	1.68
	8.2	3949	2143	327.8	233.0	1.90
#4	5.0	867	1832	398.3	238.8	0.29
	5.5	1149	2678	401.8	231.0	0.31
	6.0	1178	2172	372.5	221.5	0.41
	6.6	1471	1776	353.3	231.0	0.72
	7.1	1702	1436	353.5	227.8	1.12
	7.7	2016	1270	471.0	233.3	1.49
	8.2	2401	1255	327.8	233.0	2.03

Table designations:

$I_{pH(385)}$ and $I_{pH(465)}$ – fluorescence intensities of sfpHluorine at 385 nm and 465 nm excitation wavelengths, respectively (with 512 nm emission wavelength).

$I_{a(385)}$ and $I_{a(465)}$ - autofluorescence of yeast cells at 385 nm and 465 nm excitation wavelengths, respectively (with 512 nm emission wavelength).

$R_{385/465}$ – corrected ratio of fluorescence intensities at 385 nm and 465 nm.

(See Experimental procedures, section Measurements of cytosolic pH for detailed explanation.)

Table S12. The effect of osmotic stressors on intracellular pH of the [*psi⁻ pin⁻*] cells. (Data for Fig. 3C.)

Treatment	Time, min	Mean pH	SD	p^*
No stress	5	7.46	0.02	NA**
	25	7.32	0.06	NA
1M KCl	5	7.27	0.09	0.02
	25	7.14	0.05	0.01
2.7M sorbitol	5	7.25	0.16	0.13
	25	6.77	0.09	0.001
1.5M sorbitol	5	7.08	0.04	0.0001
	25	6.88	0.25	0.04
1M NaCl	5	7.36	0.06	0.04
	25	7.19	0.07	0.07
2M glycerol	5	7.03	0.05	0.0002
	25	6.94	0.02	0.0006

Each experiment was performed in three biological replicates.

* Probability of null hypothesis, suggesting random variation.
** NA – not applicable.

Table S13. YFP fluorescence at various pH levels. (Data for Fig. 3E.)

pH	Emission (relative to pH 8.0) in cells from cultures			Mean	SD
	1	2	3		
3.0	0.080	0.098	0.089	0.089	0.009
4.0	0.109	0.100	0.114	0.108	0.008
5.0	0.090	0.108	0.106	0.101	0.010
6.0	0.414	0.411	0.381	0.402	0.018
7.0	0.870	0.997	0.781	0.882	0.109

Table S14. Dissolution of Sup35N-YFP and Sup35NM-YFP assemblies formed in [*pin*⁻] (but not in [*PIN*⁺]) background by 1,6-hexanediol (1,6-HD). (Data for Fig. 4A.)

Prion composition	Protein	Treatment	Cells with assemblies		Total
			Number	% ± SE	
[*psi*⁻ *pin*⁻]	Sup35N-YFP	1M KCl	641	33.11 ± 1.07	1936
		1,6-HD (after 1M KCl)	2	0.16 ± 0.11	1243
	Sup35NM-YFP	1M KCl	322	7.83 ± 0.42	4112
		1,6-HD (after 1M KCl)	0	0.0 + 0.08	1252
	Sup35NM-YFP	None	177	9.78 ± 0.70	1809
		1,6-HD	0	0.00 + 0.05	2083
[*psi*⁻ *PIN*⁺]	Sup35N-YFP	None	56	36.10 ± 6.42	155
		1,6-HD	128	36.70 ± 4.26	349
	Sup35NM-YFP	None	57	19.30 ± 5.23	295
		1,6-HD	74	24.32 ± 4.99	304

Table S15. Effect of 1,6-HD on condensates after various periods of osmotic stress. (Data for Fig. 4B.)

Protein	Treatment	Time, hrs	Cells with assemblies			Total
			Number	%	SE	
Sup35N-YFP	1M KCl	0	223	18.1	1.1	1233
		0.5	444	43.4	1.6	1022
		1	413	45.9	1.7	899
		2	272	50.6	2.2	538
		3	266	49.4	2.2	538
		4	386	49.4	1.8	781
		5	266	49.4	2.2	538
		6	456	52.9	1.7	862
		24	1118	61.3	1.1	1823
		48	1150	61.3	1.1	1876
	1,6-HD after KCl	0	0	0.0	0.1	682
		0.5	0	0.0	1.6	62
		1	1	0.2	0.2	437
		2	28	6.8	1.2	414
		3	53	15.7	2.0	338
		4	33	25.4	0.2	130
		5	34	29.3	4.2	116
		6	138	35.8	2.4	385
		24	438	55.2	1.8	794
		48	167	54.9	2.9	304
Sup35NM-YFP	1M KCl	0	115	18.5	1.6	622
		0.5	603	41.8	1.3	1444
		1	252	35.0	1.8	714
		2	327	38.2	1.7	857
		3	467	41.4	1.5	1128
		4	322	41.0	1.8	785
		5	418	37.1	1.4	1127
		6	424	37.6	1.4	1127
		24	80	19.7	2.0	407
		48	68	12.9	1.5	526
	1,6-HD after KCl	0	0	0	0.5	209
		0.5	0	0	0.2	545
		1	0	0	0.3	388
		2	0	0	0.2	601
		3	0	0	0.2	508
		4	0	0	0.4	272
		5	0	0	0.2	469
		6	0	0	0.2	549
		24	3	0.5	0.3	566
		48	7	3.2	1.2	222

Table S16. Formation of Sup35N-YFP and Sup35NM-YFP condensates in the absence of Hsp104. (Data for Fig. 5D.)

Strain	Protein	Treatment	Cells with condensates		Total
			Number	% ± SE	
WT	Sup35N-YFP	None	234	9.6 ± 0.6	2438
		1M KCl	1283	43.9 ± 0.9	2919
	Sup35NM-YFP	None	54	1.9 ± 0.3	2766
		1M KCl	348	16.7 ± 0.8	2089
*hsp104*Δ	Sup35N-YFP	None	147	10.6 ± 0.8	1389
		1M KCl	557	46.5 ± 1.4	1198
	Sup35NM-YFP	None	133	4.8 ± 0.4	2761
		1M KCl	284	26.3 ± 1.3	1081

Table S17. Formation of assemblies by heterologous Sup35NM domains in the [*psi*[-] *pin*[-]] cells of *S. cerevisiae* after 24-hr overproduction. (Data for Fig. 6B.)

Treatment	Sup35NM origin	Cells with assemblies			Total
		Filaments	Puncta	% ± SE	
None	*S. cerevisiae*	0	1	1.5 ± 1.4	69
	S. paradoxus	0	0	0.0 + 1.0	96
	S. uvarum	0	1	0.6 ± 0.6	174
	N. castellii	0	5	4.2 ± 1.8	119
	O. methanolica	1	3	4.0 ± 1.9	101
1M KCl	*S. cerevisiae*	0	42	36.5 ± 4.5	115
	S. paradoxus	0	38	16.0 ± 2.4	238
	S. uvarum	0	108	53.7 ± 3.5	201
	N. castellii	0	69	45.1 ± 4.0	153
	O. methanolica	5	80	59.0 ± 4.0	144
1,6-HD after 1M KCl	*S. cerevisiae*	0	0	0.0 + 1.0	98
	S. paradoxus	0	0	0.0 + 1.5	64
	S. uvarum	0	0	0.0 + 1.2	83
	N. castellii	0	0	0.0 + 1.5	62
	O. methanolica	2	0	4.7 ± 3.0	43

Table S18. Formation of Sup35(N/NM)$_{Om}$-YFP assemblies in [*pin*$^{-}$] strain containing Sup35NM$_{Om}$-C$_{Sc}$ protein. (Data for Fig. S3B.)

Protein	Treatment	Cells with condensates		Cells with filaments		Total
		Number	% ± SE	Number	% ± SE	
Sup35N$_{Om}$-YFP	No stress	68	3.5 ± 0.4	18	0.9 ± 0.2	1929
	1M KCl	861	45.8 ± 1.2	14	0.8 ± 0.2	1841
Sup35(NM)$_{Om}$-YFP	No stress	1	0.04 ± 0.1	6	0.3 ± 0.1	2297
	1M KCl	460	18.5 ± 0.8	13	1.5 ± 0.2	2584

Table S19. Formation of various types of assemblies by Sup35(N/NM)$_{Om}$-YFP in [*psi*$^{-}$ *pin*$^{-}$] cells at background levels of Cu^{++}. (Data for Fig. 6G.)

Protein	Treatment	Cells with assemblies			Total
		Filaments	Puncta	% ± SE	
Sup35N$_{Om}$-YFP	None	3	2	0.5 ± 0.2	1026
	1M KCl	1	27	3.9 ± 0.7	719
Sup35(NM)$_{Om}$-YFP	None	0	1	0.2 ± 0.2	574
	1M KCl	0	6	1.0 ± 0.4	623

Table S20. Frequencies of Ade^{+} colonies after 24-hr osmotic stress in [*psi*$^{-}$ *pin*$^{-}$] cultures overexpressing Sup35N-HA. (Data for Fig. 7B.)

Conditions	Frequency of Ade^{+} per 10^4 cells				
	Clone 1	Clone 2	Clone 3	Clone 4	Mean ± SD
Starting culture	0.08	0.17	0.19	0.27	0.18 ± 0.08
H_2O	1.37	2.16	0.51	0.51	1.14 ± 0.79
1M KCl	2.39	3.93	0.41	0.87	1.90 ± 1.60
2M KCl	43.56	29.56	41.00	18.70	33.21 ± 11.43

Table S21. Curability of Ade+ colonies induced in [*psi*- *pin*-] cultures overexpressing Sup35N-HA. (Data for Fig. 7C.)

Treatment	Colonies		
	Curable*	% curable	Total
H_2O	47	100	47
1M KCl	44	100	44
2M KCl	48	100	48

*By 5 mM GuHCl.

Table S22. Frequencies of Ade+ colonies after 24-hr osmotic stress in the [*pin*- *psi*-] strain with Sup35(NM)$_{Om}$-C$_{Sc}$ protein. (Data for Fig. 8B.)

Conditions	Frequency of Ade+ per 10^4 cells				
	Culture 1	Culture 2	Culture 3	Culture 4	Mean ± SD
H_2O	1.53	4.13	0.88	2.78	2.33 ± 1.44
1M KCl	7.35	8.33	2.17	6.46	6.08 ± 2.72
2M KCl	6.24	10.74	2.78	11.27	7.76 ± 4.01

Table S23. Frequencies of [*PSI*+] colonies after 24-hr osmotic stress in the [*psi*- *pin*-] strain with Sup35(NM)$_{Om}$-C$_{Sc}$ after overproduction of Sup35(N/NM)$_{Om}$-YFP. (For Fig. 8C.)

Protein	Conditions	Frequency of Ade+ per 10^4 cells					
		Culture					Mean ± SD
		1	2	3	4	5	
Sup35N$_{Om}$-YFP	H_2O	0.2	0.4	0.2	0.3	0.4	0.3 ± 0.1
	1M KCl	1.5	0.7	0.7	25.4	1.4	5.9 ± 10.9
	2M KCl	80.2	35.4	41.3	29.7	26.8	42.7 ± 21.7
Sup35(NM)$_{Om}$-YFP	H_2O	2.1	3.2	1.6	1.1	NA	2.0 ± 0.9
	1M KCl	15.5	19.7	10.5	14.6	NA	15.1 ± 3.8
	2M KCl	33.3	72.3	41.3	35.8	NA	45.7 ± 18.1

Table S24. Curability of Ade$^+$ colonies induced in strain with Sup35(NM)$_{Om}$-C$_{Sc}$ protein. (Data for Fig. 8D.)

Treatment	Colonies		
	Curable*	% curable	Total
H_2O	50	78.1	64
1M KCl	36	73.5	49
2M KCl	50	86.2	58

*By 5 mM GuHCl.

Table S25. Curability of Ade$^+$ colonies induced in strain with Sup35(NM)$_{Om}$-C$_{Sc}$, overproducing Sup35(N/NM)$_{Om}$-YFP. (Data for Fig. 8D.)

Protein	Treatment	Colonies		
		Curable*	% curable	Total
Sup35N$_{Om}$-YFP	H_2O	10	100	10
	1M KCl	10	100	10
	2M KCl	10	100	10
Sup35(NM)$_{Om}$-YFP	H_2O	0	0	10
	1M KCl	4	33	12
	2M KCl	12	100	12

*By 5 mM GuHCl.

Table S26. Viability of *S. cerevisiae* isolates with Sup35(NM)$_{Om}$-C$_{Sc}$ protein after 24-hr osmotic stress. (Data for Fig. 8E.)

Prion	Treatment	Proportion of viable cells relative to starting point					
		1	2	3	4	5	Mean ± SD
[*psi*$^-$]	H_2O	1.02	0.83	0.38	0.90	0.77	0.78 ± 0.24
	1M KCl	0.24	0.17	0.27	0.10	0.05	0.17 ± 0.09
	2M KCl	0.34	1.00	0.26	0.20	0.13	0.39 ± 0.35
Weak [*PSI*$^+$]	H_2O	0.65	2.31	0.48	1.09	0.79	0.85 ± 0.22
	1M KCl	0.13	0.37	0.17	0.08	0.05	0.09 ± 0.04
	2M KCl	0.27	1.00	0.26	0.16	0.12	0.19 ± 0.08
Strong [*PSI*$^+$]	H_2O	0.62	0.86	0.68	1.05	1.30	0.90 ± 0.28
	1M KCl	0.52	0.15	0.12	0.09	0.13	0.20 ± 0.18
	2M KCl	0.60	0.76	0.35	0.38	0.46	0.51 ± 0.17

Table S27. Conversion of *O. methanolica* condensates to the amyloid filaments in [*pin*$^-$ *psi*$^-$] *S. cerevisiae* cells incubated with 1M KCl. (Data for Fig. 9B.)

Protein	Time, min	Number of cells				
		with filaments	% ± SE	with condensates	% ± SE	Total
$Sup35N_{Om}$-YFP	5	21	3.7 ± 0.8	173	30.7 ± 1.9	565
	100	161	28.5 ± 1.9	35	6.2 ± 1.0	564
$Sup35(NM)_{Om}$-YFP	5	9	2.4 ± 0.8	111	29.5 ± 2.4	376
	100	170	29.2 ± 1.9	12	2.1 ± 0.6	583

Table S28. Comparison of protein levels at background levels (3μM) of Cu^{++} using densitometry. (Data for Fig. S5D.)

Protein	Number of repeats	Range of variation	Mean	SD
$Sup35(NM)_{Sc}$-YFP	13	0.754 – 1.246	**1.000**	0.112
$Sup35N_{Sc}$-YFP	5	0.992 – 1.421	**1.182**	0.192
$Sup35(NM)_{Om}$-YFP	6	0.846 – 1.411	**1.132**	0.188
$Sup35N_{Om}$-YFP	6	0.698 – 1.503	**1.060**	0.283
$Sup35_{Sc}$	8	0.023 – 0.152	**0.064**	0.042
$Sup35_{Sc}$-GFP	3	0.030 – 0.044	**0.037**	0.007

Table S29. Dependence of YFP fluorescence on the culture density. (Data for Fig. S5E.)

OD_{600}	Fluorescence intensities of cells grown in the presence of		Autofluorescence
	No extra Cu^{++}	Extra Cu^{++}	
0.05	12046	20070	11343
0.1	24746	81312	23295
0.2	48428	266494	40245
0.3	78440	505513	55210
0.4	106565	786225	69209
0.5	141730	1216363	83125
1	323929	3033899	108660
2	553672	6793043	130688
4	1022642	12749313	155835
6	1511787	18358714	193531
8	1842872	24329163	219569
10	2289637	30266615	218955

Table S30. Comparison of protein levels at low and high levels of Cu^{++} using fluorescence measurements. (Data for Fig. S5F.)

Protein	Extra Cu^{++}	Number of repeats	Range of variation	Mean	SD
$Sup35(NM)_{Sc}$-YFP	No	5	0.75 – 1.28	**1.00**	0.28
$Sup35N_{Sc}$-YFP	No	6	0.74 – 1.44	**1.19**	0.42
$Sup35(NM)_{Om}$-YFP	No	5	0.71 – 1.53	**1.22**	0.39
$Sup35N_{Om}$-YFP	No	6	0.73 – 1.98	**1.15**	0.45
$Sup35(NM)_{Sc}$-YFP	Yes	5	12.76 – 15.85	**14.15**	1.43
$Sup35N_{Sc}$-YFP	Yes	6	6.73 – 9.03	**7.83**	0.85
$Sup35(NM)_{Om}$-YFP	Yes	6	6.86 – 14.35	**9.16**	2.78
$Sup35N_{Om}$-YFP	Yes	6	4.68 – 9.23	**6.21**	1.61

Table S31. Comparison of amino acid compositions and aggregation properties of Sup35N domains from different yeast species.

Origin of Sup35N	Length, aa	Glutamine (Q) residues		Asparagine (N) residues		Formation of filaments in *S. cerevisiae*	
		Number	%	Number	%	[*psi*⁻ *pin*⁻]	[*psi*⁻ *PIN*⁺]
S. cerevisiae	123	35	33.3	20	16.2	-	+
S. paradoxus	123	35	28.5	19	15.4	-	+
S. uvarum	116	34	29.3	17	14.7	-	+
N. castellii	130	52	40.0	15	11.5	-	+
O. methanolica	161	30	18.6	42	26.1	+	+

Table S32. Yeast strains used in this study.

Strain name	Prion composition	Strain origin	Genotype
GT409	[psi^- pin^-]	GT81	*MAT***a** *ade1-14 his3 leu2 lys2 trp1 ura3*
GT159	[psi^- PIN^+]	GT81	*MAT***a** *ade1-14 his3 leu2 lys2 trp1 ura3*
GT81-1C	[PSI^+ PIN^+]	GT81	*MAT***a** *ade1-14 his3 leu2 lys2 trp1 ura3*
GT1569	[psi^- pin^-]	GT81	*MAT***a** *ade1-14 his3 leu2 lys2 trp1 ura3 hsp104Δ::ura3*
GT225-6B	[psi^- pin^-]	GT81	*MATα ade1-14 his3 leu2 lys2 trp1 ura3 sup35Δ::HIS3* [$SUP35NM_{Om}$-C_{Sc}]
7-74-D694	[PSI^+ PIN^+]	74-D694	*MAT***a** *ade1-14 his3 leu2 trp1 ura3*
AB190	[psi^- pin^-]	74-D694	*MAT***a** *ade1-14 his3 leu2 trp1 ura3 sup35-ΔNM*
SY831	[PSI^+ PIN^+]	74-D694	*MAT***a** *ade1-14 his3 leu2 trp1 ura3 SUP35N::mCherry::MC*
GT2508	[psi^- pin^-]	74-D694	*MAT***a** *ade1-14 his3 leu2 trp1 ura3 SUP35N::mCherry::MC*
GT2518	[psi^- pin^-]	779-6A	*MATa ade2-1 his3-Δ202 leu2-Δ1 trp1-Δ6, ura3-52 SUP35N::GFP::MC kar1-1 SUQ5*

Table S33. *Saccharomyces cerevisiae – Escherichia coli* shuttle plasmids used in this study.

Plasmid name	Plasmid type	Yeast marker	Promoter	Expression cassette	Origin
pRS316	*CEN*	*URA3*	-	-	(83)
pRS315	*CEN*	*LEU2*	-	-	(83)
pmCUP1-LEU2	*CEN*	*LEU2*	P_{CUP1}	-	This study
pmCUP1	*CEN*	*URA3*	P_{CUP1}	-	(80)
pmCUP1-YFP	*CEN*	*LEU2*	P_{CUP1}	*YFP*	This study
pmCUP1-Sup35N-HA	*CEN*	*URA3*	P_{CUP1}	*SUP35N-HA*	(59)
p316CUP1-Sup35NM$_{Sc}$-YFP	*CEN*	*URA3*	P_{CUP1}	*SUP35NM-YFP*	This study
p316CUP1-Sup35N$_{Sc}$-YFP	*CEN*	*URA3*	P_{CUP1}	*SUP35NM-YFP*	This study
pmCUP1-Sup35NM-YFP	*CEN*	*LEU2*	P_{CUP1}	*SUP35NM-YFP*	(85)
pUC-SUP35NMPmCSc	*CEN*	*URA3*	P_{Sup35}	*SUP35(NM)*$_{Om}$-*SUP35C*$_{Sc}$	(56)
pmCUP1-Sup35NM$_{Sp}$-YFP	*CEN*	*LEU2*	P_{CUP1}	*SUP35(NM)*$_{Sp}$-*YFP*	This study
pmCUP1-Sup35NM$_{Su}$-YFP	*CEN*	*LEU2*	P_{CUP1}	*SUP35(NM)*$_{Su}$-*YFP*	This study
pmCUP1-Sup35NM$_{Nc}$-YFP	*CEN*	*LEU2*	P_{CUP1}	*SUP35(NM)*$_{Nc}$-*YFP*	This study
pmCUP1-Sup35NM$_{Om}$-YFP	*CEN*	*LEU2*	P_{CUP1}	*SUP35(NM)*$_{Om}$-*YFP*	This study
pmCUP1-Sup35N$_{Om}$-YFP	*CEN*	*LEU2*	P_{CUP1}	*SUP35N*$_{Om}$-*YFP*	This study
pmCUP-Sup35NMSbay-sGFP	*CEN*	*URA3*	P_{CUP1}	*SUP35NM*$_{Su}$-*sGFP*	(58)
pmCUP-NMSPsGFP	*CEN*	*URA3*	P_{CUP1}	*SUP35NM*$_{Sp}$-*sGFP*	(58)
pmCUP-Sup35M-CFP	*CEN*	*LEU2*	P_{CUP1}	*SUP35M*$_{Sc}$-*YFP*	This study
p426MET25_sfpHluorin (MRV55)	2μ	*URA3*	P_{MET25}	*sfpHluorin*	(51)

Table S34. Oligonucleotide primers used in this study.

Name	Sequence 5'-3'	Created plasmid
F-BamHI-Sup35$_{S.castellii}$	CTGA**GGATCC**atgtccaatccataccaaggtaatgg	pmCUP1-Sup35NM$_{Nc}$-YFP
R-Sup35$_{S.castellii}$-SacII	CTAG**CCGCGG**atcattaacaacttcttcatcgactt	
F-NotI-YFP	TTAGT**GCGGCCGC**tatgagtaaaggagaagaac	pmCUP1-YFP
R-YFP-SacI	ATT**GAGCTC**tcatttgtatagttcatccatgcc	
F-BamHI-Sup35$_{O.methanolica}$	ATCA**GGATCC**aaaatgtctcaagatcaac	pmCUP1-Sup35NM$_{Om}$-YFP
R-Sup35$_{O.methanolica}$-XbaI	GATT**TCTAGA**ttgaccggaacc	
R-Sup35N$_{O.methanolica}$-XbaI	GATT**TCTAGA**ttgaccggaaccggtc	pmCUP1-Sup35N$_{Om}$-YFP

Restriction sites introduced into the sequences are shown in bold, while sequence homologues to the target are shown in lowercase.

Supporting Information Video Descriptions

Video S1. The 3D reconstruction of cells with Sup35N-YFP biomolecular condensates. The movie depicts *S. cerevisiae* [*pin*$^-$ *psi*$^-$] *sup35-ΔNM* cells with overproduced Sup35N-YFP (after growth in medium with addition of 100 μM $CuSO_4$), incubated in 1M KCl for 5 min. Images were captured using confocal microscopy. 3D reconstruction, demonstrating the round shape of condensates, was created using FIJI software (RRID:SCR_002285) (87).

Video S2. The 3D reconstruction of cells with Sup35NM-YFP biomolecular condensates. The movie depicts *S. cerevisiae* [*pin*$^-$ *psi*$^-$] *sup35-ΔNM* cells with overproduced Sup35NM-YFP (after growth in medium with addition of 100 μM $CuSO_4$), incubated at 1M KCl for 5 min. Images were captured using confocal microscopy. 3D reconstruction, demonstrating the round shape of condensates, was created using FIJI software (87).

Video S3. FRAP of Sup35NM-GFP condensates formed in response to osmotic stress in [*psi*$^-$ *pin*$^-$] cells. Condensates, induced in *S. cerevisiae* [*psi*$^-$ *pin*$^-$] cells with overproduced Sup35NM-GFP (after growth in the medium with addition of 100 μM $CuSO_4$ for 24 hrs) by incubation with 1M KCl for 1 hr were photobleached using a high power laser. Twenty frames were collected prior to bleaching, followed by point bleaching for 1 frame, and collection of 400 frames after bleaching (with 1 frame per every 0.518 sec). A representative movie created with FIJI software demonstrates fast recovery of fluorescence within the bleached spot (87). For static images and graphs, see Fig. 4CD.

Video S4. FRAP of Sup35NM-GFP amyloid aggregates in [*PSI*$^+$ *PIN*$^+$] cells. Aggregates produced in the *S. cerevisiae* [*PSI*$^+$ *PIN*$^-$] cells, overexpressing Sup35NM-GFP with 100 µM $CuSO_4$ during 24 hrs were photobleached using a high power laser. Twenty frames were collected prior to bleaching, followed by point bleaching for 1 frame and collection of 400 frames (one per every 0.518 sec) after bleaching. The observed fluorescence recovery within the bleached spot was negligible, as depicted in a representative movie generated using FIJI software (87). For static images and graphs, see Fig. 4CD.

Video S5. FRAP of Sup35N-GFP condensates formed in response to osmotic stress in [*psi*$^-$ *pin*$^-$] cells. Sup35NM-GFP condensates induced in *S. cerevisiae* [*psi*$^-$ *pin*$^-$] cells, grown in the presence of 100 µM $CuSO_4$ for 24 hrs by incubation with 1M KCl for 1 hr were photobleached using high power laser. 20 frames were collected prior to bleaching, followed by point bleaching for one frame, and collection of 400 frames after bleaching (with 1 frame per every 0.518 sec). A representative movie created with FIJI software (87) demonstrates fast recovery of fluorescence within the bleached spot. For static images and graphs, see Fig. 4CD.

Video S6. Conversion of condensates into amyloid filaments during osmotic stress in yeast cells overproducing Sup35N$_{Om}$-YFP. Representative time-lapse fluorescence microscopy (FM) video of *S. cerevisiae* [*psi*$^-$ *pin*$^-$] *sup35-ΔNM* cells with Sup35N-YFP from *O. methanolica* (induced in the medium with 100 µM $CuSO_4$), incubated in the presence of 1M KCl. Video was created using FIJI software (87), with one frame per each 10 min, for the total time period of 90 min. Conversion of round condensates into filamentous amyloid aggregates can be observed. For static images, see Fig. 9A.

Video S7. Conversion of condensates into amyloid filaments during osmotic stress in yeast cells overproducing Sup35(NM)$_{Om}$-YFP. Representative time-lapse FM video of *S. cerevisiae* [*psi*$^{-}$ *pin*$^{-}$] *sup35-ΔNM* cells with Sup35NM-YFP protein from *O. methanolica* (induced in the medium with 100 µM $CuSO_4$), incubated in the presence of 1M KCl. Video was created using FIJI software (87), with one frame every 10 min, for a total time period of 90 min. Conversion of round condensates into filamentous amyloid aggregates can be observed. For static images, see Fig. 9A.

Video S8. Conversion of Sup35N$_{Om}$-YFP condensates into amyloid filaments during osmotic stress without overproduction. Representative time-lapse FM video of *S. cerevisiae* [*psi*$^{-}$ *pin*$^{-}$] *sup35-ΔNM* cells with Sup35N-YFP protein from *O. methanolica* (grown in medium without addition of extra Cu^{++}), incubated in the presence of 1M KCl. Video was created using FIJI software (87), with one frame per each 10 min, for a total time period of 150 min. Conversion of round condensates into filamentous amyloid aggregates can be observed. For static images, see Fig. 9C.

Video S9. Conversion of Sup35(NM)$_{Om}$-YFP condensates into amyloid filaments during osmotic stress without overproduction. Representative time-lapse FM video of *S. cerevisiae* [*psi*$^{-}$ *pin*$^{-}$] *sup35-ΔNM* cells with Sup35NM-YFP protein from *O. methanolica* (grown in medium without addition of extra Cu^{++}), incubated in the presence of 1M KCl. Video was created using FIJI software (87), with one frame every 10 min, for a total time period of 170 min. Conversion of round condensates into filamentous amyloid aggregates can be observed. For static images, see Fig. 9C.

Supporting Information Dataset Descriptions

Dataset S1. pH dependence of excitation spectra of sfpHluorin expressed in yeast. The fluorescence of sfpHluorin expressed in the [*psi*$^-$ *pin*$^-$] *sup35-ΔNM S. cerevisiae* cells was measured at an emission wavelength of 512 nm with different excitation wavelengths, ranging in 5 nm increments from 355 nm to 475 nm. Four independent cultures were tested. Resulting graphs are shown on Fig. 2A.

Dataset S2. FRAP data for the Sup35N/NM-GFP assemblies. The dataset contains all raw measurements of fluorescent intensities for all samples before (20 frames) and after (400 frames) bleaching, as well as measurements of the background fluorescence under the same conditions, for the experiments shown on Fig. 4CD and in Videos S3, S4 and S5.
